# Polymeric Composite Thin Films Deposited by Laser Techniques for Antimicrobial Applications—A Short Overview

**DOI:** 10.3390/polym17152020

**Published:** 2025-07-24

**Authors:** Anita Ioana Visan, Irina Negut

**Affiliations:** National Institute for Laser, Plasma and Radiation Physics, 409 Atomistilor Street, P.O. Box MG 36, 077125 Magurele, Romania; anita.visan@inflpr.ro

**Keywords:** antimicrobial thin films, polymeric composites, laser deposition techniques, pulsed laser deposition, matrix-assisted pulsed laser evaporation

## Abstract

Polymeric composite thin films have emerged as promising antimicrobial materials, particularly in response to rising antibiotic resistance. This review highlights the development and application of such films produced by laser-based deposition techniques, notably pulsed laser deposition and matrix-assisted pulsed laser evaporation. These methods offer precise control over film composition, structure, and thickness, making them ideal for embedding antimicrobial agents such as metal nanoparticles, antibiotics, and natural compounds into polymeric matrices. The resulting composite coatings exhibit enhanced antimicrobial properties against a wide range of pathogens, including antibiotic-resistant strains, by leveraging mechanisms such as ion release, reactive oxygen species generation, and membrane disruption. The review also discusses critical parameters influencing antimicrobial efficacy, including film morphology, composition, and substrate interactions. Applications include biomedical devices, implants, wound dressings, and surfaces in the healthcare and food industries.

## 1. Introduction

The growing concern over antibiotic resistance has spurred extensive research into novel antimicrobial strategies, particularly the development of polymeric composite thin films [1]. These films are produced using advanced deposition techniques and show promise in various applications, including biomedical devices, food packaging, and water purification. The unique properties of polymeric materials, such as flexibility and biocompatibility, combined with the precision of laser-based methods, pave the way for creating effective antimicrobial surfaces [1,2]. Biomedical devices increasingly require flexibility to enhance patient comfort, diagnostic capabilities, and treatment efficacy [3]. This is crucial for a range of applications, including wearable sensors like smart bandages [4] and electronic skin patches [5], which need to adapt to skin movements. Similarly, various implantable devices, such as neuroprosthetics [6], cardiac implants [7], retinal implants [8], and gastrointestinal/urological devices [9], rely on flexibility to interact harmoniously with soft biological tissues and endure constant internal movements. Additionally, flexible endoscopes [10] and catheters [11] benefit from this property by improving maneuverability and minimizing tissue trauma, while flexible patches and implants are vital for conformable drug delivery. Essential mechanical properties for these devices include a low Young’s modulus for softness, a high elastic limit, and high ultimate strain, allowing them to bend, stretch, and twist without functional or structural compromise [12].

Thin film deposition techniques utilizing lasers have emerged as versatile tools for fabricating polymeric composite coatings with tailored antimicrobial properties. Pulsed laser deposition (PLD) allows for precise control over film thickness and stoichiometry, enabling the incorporation of various antimicrobial agents within a polymer matrix [13].

Matrix-assisted pulsed laser evaporation (MAPLE) is particularly gentle for delicate biomolecules, facilitating the deposition of active antimicrobial compounds without degradation [14].

These techniques enable the integration of a diverse array of antimicrobial agents, including metallic nanoparticles (NPs) (e.g., silver [15], gold [16]), metal oxides (e.g., titanium dioxide [17]), zinc oxide [18]), and even organic antimicrobial compounds, within the polymeric matrix. The processes induced by laser technology can also generate unique interfacial interactions between the polymer and the antimicrobial agent, thereby potentially enhancing the stability and sustained release of the active component, which in turn improves the long-term antimicrobial efficacy of the film [19].

This review offers an overview of polymeric composite thin films fabricated using laser deposition techniques for antimicrobial applications. We will cover the fundamental characteristics, including common polymeric matrices and various antimicrobial agents, their mechanisms, and methods of integration. A significant portion will detail laser-based deposition techniques like PLD and MAPLE, focusing on how process parameters influence film properties and optimize antimicrobial performance. We will also explore characterization techniques that link film composition to antimicrobial activity. Subsequent sections will assess the antimicrobial efficacy of these films against bacteria, fungi, and viruses, evaluating diverse measurement methodologies. We will highlight factors that affect performance, including agent type and concentration, polymer properties, and surface characteristics.

We will also address current challenges and future prospects, including biocompatibility, stability, and scalability. This aims to stimulate further research in practical, sustainable antimicrobial solutions.

**Figure 1:**
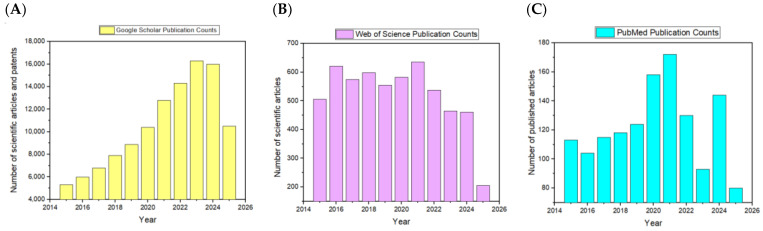
Histograms that illustrate the number of publications related to specific research areas over the last decade (2015–2025). Each histogram corresponds to a different search query: (**A**). “antimicrobial polymeric laser coatings” on Google Scholar (including scientific articles and patents); (**B**). “Polymeric thin films” on Web of Science; and (**C**). “laser polymeric coatings” on PubMed. Distinct trends emerge: a niche or mature biomedical application for “polymeric coatings laser” (**C**), explosive growth in the commercially relevant “polymeric laser coatings antimicrobial” field (**A**), and a stable, foundational role for “polymeric thin films” (**B**). This comprehensive approach, utilizing multiple database sources—PubMed for a biomedical lens, Web of Science for core academic rigor, and Google Scholar for a broader technological and commercial spectrum, including patents—captures the full range of scientific and technological activity, from fundamental research to applied innovation and intellectual property development. While specific trends vary considerably across different research foci and databases, the general landscape of research involving polymers and lasers appears robust, particularly in applied domains.

The analysis of publication trends across PubMed NIH, Google Scholar, and Web of Science provides a nuanced understanding of the broader research landscape concerning polymeric and laser technologies, as illustrated in Figure 1.

While the integration of antimicrobial agents into polymeric matrices for thin film applications has been covered in several review articles [20,21] there is a notable gap in the comprehensive review literature specifically focusing on polymeric composite thin films deposited exclusively by laser-based techniques for antimicrobial applications. Existing reviews often discuss a broader range of antimicrobial thin films [22,23] encompassing various deposition methods like sputtering [24], chemical vapor deposition [25], and solution processing [24], with only limited coverage of laser deposition techniques such as PLD and MAPLE [26]. These reviews offer valuable insights into the types of antimicrobial agents (e.g., metal NPs [27], organic compounds [28] incorporated into polymeric films, and their mechanisms of action [29]). They also discuss the antimicrobial efficacy of these films against different microorganisms and their potential applications in diverse fields like biomedicine and food packaging [23]. Moreover, the global market for antimicrobial coatings is projected to reach USD 7.5 billion by 2027, primarily driven by the increasing challenge of antibiotic resistance and a growing demand for infection-resistant surfaces across the healthcare and food industries [30]. To meet this demand and for further development, key needs include the following: (i) enhancing scalability by transitioning from lab characteristic laser techniques (like MAPLE) to industrial production; (ii) improving cost-effectiveness through the reduction in reliance on expensive materials such as Ag NPs via hybrid composites; (iii) ensuring regulatory compliance by meeting stringent biocompatibility standards like ISO 10993 for medical applications; and (iv) achieving multifunctionality by combining antimicrobial properties with mechanical durability and stimuli-responsiveness [31]. Thus, a dedicated review that specifically consolidates the advancements, challenges, and future directions concerning the use of laser-based techniques for fabricating antimicrobial polymeric composite thin films, emphasizing the unique advantages and the intricate process-structure-property relationships associated with these methods, would offer a more targeted and in-depth understanding of this emerging field.

## 2. Laser Deposition Techniques for Antimicrobial Polymeric Composite Thin Films

Laser deposition techniques have become powerful tools for creating thin films with precise control over their composition, thickness, morphology, and even spatial patterns. For antimicrobial polymeric composite thin films, these methods offer unique advantages in integrating diverse antimicrobial agents within various polymer matrices. The high energy density and short pulse durations of lasers enable the controlled ablation of materials and the subsequent deposition of films with customized properties. This section will detail the primary laser deposition techniques used to fabricate antimicrobial polymeric composite thin films, their operating principles, key parameters affecting film growth, and their specific applications in creating antimicrobial surfaces.

Table 1 summarizes various laser deposition techniques and the advantages and disadvantages of each laser technique for the deposition of antimicrobial polymeric thin films.

The choice of laser deposition technique for fabricating antimicrobial polymeric composite thin films depends on several factors, including the nature of the polymer and the antimicrobial agent, the desired film properties (thickness, morphology, uniformity, spatial pattern), the substrate material, and the scalability and cost-effectiveness of the process [34,35].

### 2.1. Mechanical Considerations in Laser-Deposited Flexible Films

Maintaining this crucial flexibility after applying a PLD coating involves several strategic approaches [12]. Choosing inherently flexible substrates, including polyimide [36], PET [37], PDMS [38], and thin metallic foils [39], is essential to ensure mechanical compliance in flexible device applications. The PLD coating itself must be as thin as possible, ideally at the nanometer scale, as thinner films are intrinsically more flexible and less susceptible to cracking under strain [39]. The choice of coating material is also vital; intrinsically flexible materials or those tolerant to strain, such as amorphous or nanocrystalline films [40] (e.g., diamond-like carbon, certain metal oxides), are preferred. Furthermore, advanced coating architectures like graded interfaces or multilayer stacks can mitigate stress concentrations [41]. Patterning the PLD coating into serpentine, wavy, or kirigami-like geometries is another effective strategy, allowing the overall device to deform while localized segments of the coating remain relatively unstrained [42]. Optimizing PLD parameters for low-temperature deposition helps minimize thermal stresses, and carefully controlled post-deposition annealing or the use of an intermediate adhesion layer can enhance film adhesion and relieve residual stresses, further improving flexibility [43].

The required mechanical durability, specifically in terms of cycles and strain/bend parameters, is highly application-dependent and must align with the in vivo environment and regulatory standards from bodies like the FDA and ISO [44]. Biomedical devices must withstand millions of physiological movements over their lifespan; for instance, a cardiac implant experiences tens of millions of cycles annually [45]. Fatigue testing is employed to identify critical failure modes such as cracking or delamination, with specific cycle requirements ranging from thousands for wearable or temporary devices to hundreds of millions for long-term implants (e.g., 10^7^ to 10^8^ cycles for cardiovascular or orthopedic devices over 10–15 years) [46]. Strain, expressed as a percentage, defines the deformation, with brittle coatings demanding very low strains (<0.5%), while structured flexible coatings might tolerate overall device strains of 5–10% or more, provided local coating strains remain minimal [12]. Bending radius, evaluated by bending a sample over a mandrel, or specific angles of bending (e.g., ±30°, ±90°) are also crucial test parameters, mimicking physiological curvatures [12]. For example, PLD-coated vascular stents require high fatigue resistance to radial and axial deformations, typically tested for millions of cycles (e.g., 10^7^) at 2–5% radial strain [47]. Wearable patches, in contrast, need to withstand repeated skin movement, requiring tens to hundreds of thousands of cycles at bend radii of 5–20 mm [48]. Implantable neuroprosthetics demand millions of cycles of subtle bending and stretching at low strains, while coatings on catheters must endure thousands of cycles of complex bending and sliding during insertion and removal [49].

PLD is suitable to be applied for the deposition of a wide range of materials, including polymers and inorganic nanoparticles. It offers relatively high deposition rates and the potential for stoichiometric transfer [50,51]. However, particulate emission and achieving uniform deposition over large areas can be challenges. MAPLE is particularly advantageous for depositing delicate organic molecules and fragile polymers with minimal thermal damage. It often yields smooth films but typically has lower deposition rates and may require post-deposition solvent removal [52].

### 2.2. PLD and MAPLE

#### 2.2.1. Principle

**Figure 2:**
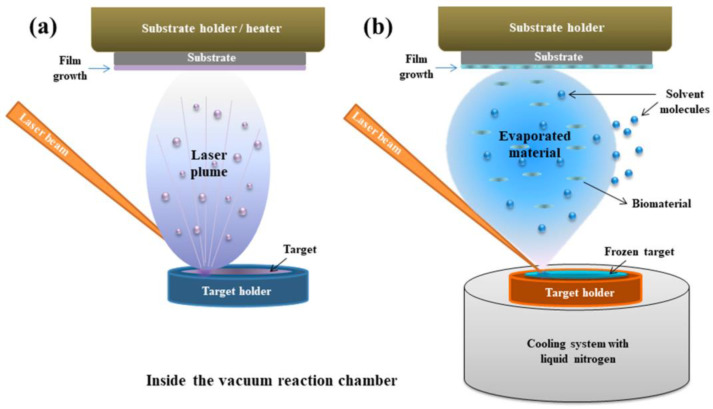
Differences between laser ablation (**a**) and evaporation (**b**) processes [58].

PLD is a versatile physical vapor deposition technique that uses a high-power pulsed laser beam to ablate material from a target and deposit it as a thin film onto a substrate in a vacuum chamber [50,51]. The fundamental process of PLD involves three key steps. First, laser ablation occurs when a focused pulsed laser beam, with high energy density, strikes a target material (Figure 2a). The absorption of this laser energy causes rapid heating, melting, vaporization, and the ejection of a plasma plume containing atoms, ions, electrons, clusters, and even microparticles from the target. A significant advantage of PLD for multi-component materials is that the composition of the ablated material closely mirrors that of the target. Second, the plasma plume expands away from the target surface. The characteristics of this plume, including its temperature, density, and the kinetic energy of its constituents, are heavily influenced by laser parameters (fluence, wavelength, pulse duration, repetition rate) and the target material’s properties [53,54,55]. Third, film deposition takes place as the expanding plasma plume travels toward a substrate, typically positioned parallel to the target. Upon reaching the substrate, the energetic species in the plume condense and nucleate, forming a thin film. The film’s growth kinetics and resulting properties (thickness, morphology, crystallinity, and stoichiometry) are affected by factors such as substrate temperature, background gas pressure (if any), and the arrival rate and energy of the depositing species [56,57].

**Figure 3:**
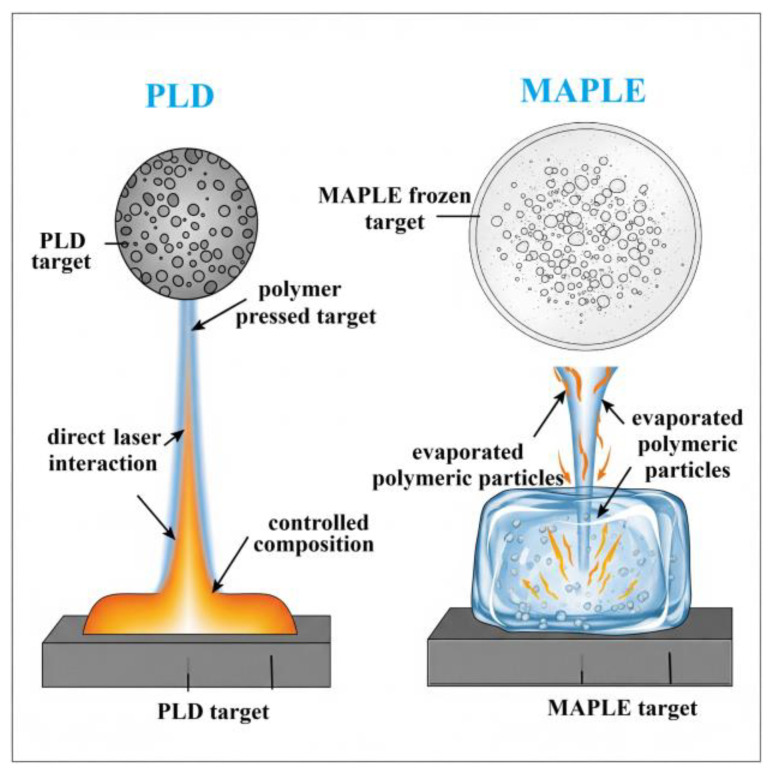
Comparative illustration of PLD and MAPLE techniques for thin film deposition. The left side depicts the PLD process, showing a solid polymer pressed into a target being ablated by a laser for direct laser interaction and controlled composition film growth. The right side illustrates the MAPLE process, featuring a MAPLE frozen target containing the dispersed polymer within a frozen matrix, from which evaporated polymeric particles are transferred.

MAPLE is another laser-based thin film deposition technique, particularly well-suited for depositing delicate organic molecules and polymers without causing significant thermal decomposition [59]. In contrast to PLD, where the laser directly ablates the target material, MAPLE involves embedding the material to be deposited (the “analyte” or active substance) in a volatile solvent matrix (Figure 3).

The MAPLE process typically involves several steps. First, for target preparation, the analyte (e.g., a polymer or an organic antimicrobial molecule) is dissolved or dispersed in a volatile solvent (the matrix) at a relatively low concentration (typically 1–5 wt%). This solution is then frozen to form a solid target [59]. Second, during laser irradiation, a pulsed laser beam, usually in the UV or visible range, is directed onto the frozen target. The laser energy is primarily absorbed by the matrix material, resulting in its rapid evaporation (Figure 2b). Third, for analyte transfer, as the matrix evaporates, the analyte molecules or NPs embedded within it are carried away and deposited onto a substrate placed above the target in a vacuum chamber. Due to the low concentration of the analyte in the matrix and the indirect ablation mechanism, the analyte experiences significantly less thermal stress compared to direct laser ablation [60]. Finally, for solvent evaporation from the film, the deposited film consists of the analyte embedded in residual matrix material. Post-deposition annealing or vacuum drying is often employed to remove the remaining solvent and obtain a pure film of the analyte [61].

#### 2.2.2. Application to Polymeric Composite Thin Films

PLD has been successfully applied to deposit polymeric thin films and, more importantly, to fabricate polymeric composite thin films incorporating antimicrobial agents. A primary challenge in the PLD of polymers is their susceptibility to thermal degradation due to the high laser energy. However, by carefully controlling laser parameters, particularly using short pulse durations (femtoseconds to nanoseconds) and optimized laser fluence, it is possible to ablate polymeric materials with minimal thermal damage and achieve stoichiometric transfer to the growing film [62].

For depositing polymeric composite thin films containing inorganic antimicrobial nanoparticles (e.g., Ag NPs, Cu NPs, TiO_2_), several PLD approaches can be adopted. One is composite target ablation, a one-step method involving ablating a target made of a mixture of the polymer and the antimicrobial agent (e.g., NPs dispersed in a polymeric matrix) [63]. While aiming for simultaneous transfer, achieving uniform nanoparticle dispersion in the deposited film can be challenging due to differing ablation thresholds and dynamics between the polymer and nanoparticles. Another approach is sequential ablation, which uses separate targets for the polymer and the antimicrobial agent (e.g., a pure metal target for metallic nanoparticles or a metal oxide target) [64]. By controlling the number of laser pulses on each target, the relative amounts of polymer and antimicrobial agent in the film can be precisely managed, offering greater flexibility in tailoring composition and potentially leading to more homogeneous distribution [65]. In some cases, reactive PLD can be employed, where a reactive background gas (e.g., oxygen) is introduced into the vacuum chamber. This allows for controlling the oxidation state of the deposited antimicrobial agent, for instance, forming metal oxide nanoparticles within the polymer film in situ [62].

MAPLE is particularly advantageous for depositing antimicrobial polymeric composite thin films containing organic antimicrobial agents, such as antibiotics or peptides, which are prone to degradation at high temperatures [66].

It also allows for the deposition of inherently fragile polymers or biomacromolecules with minimal structural damage. For the fabrication of polymeric composite thin films with inorganic nanoparticles using MAPLE, the nanoparticles are typically dispersed within a polymer solution in a volatile solvent, and this mixture is then frozen to form the target. Upon laser irradiation, the solvent evaporates, carrying the polymer and the nanoparticles to the substrate [67].

#### 2.2.3. Key Parameters Influencing PLD and MAPLE for Polymeric Composite Thin Films

Several key parameters influence the PLD process for polymeric composites. The table below (Table 2) outlines the critical deposition parameters influencing PLD and MAPLE techniques. Each parameter affects film quality, composition, morphology, or functional performance.

Laser wavelength is crucial, as the target material’s absorption coefficient at this wavelength significantly impacts ablation [74]. For polymers, UV wavelengths are often preferred due to strong absorption and efficient bond breaking, resulting in cleaner ablation. Laser fluence (energy per unit area per pulse) determines the amount of material ablated. Optimal fluence is essential for efficient material transfer while minimizing thermal damage to the polymer [61]. Pulse duration and repetition rate also play a role; short pulse durations (nanoseconds or shorter) minimize thermal diffusion into the target, leading to more congruent ablation, while the repetition rate affects the deposition rate and the kinetic energy of the ablated species [75]. Substrate temperature influences the surface mobility of depositing species, affecting film morphology, adhesion, and crystallinity [76]. For polymeric films, careful control is vital to prevent polymer degradation [77]. Background gas pressure and composition can influence plasma plume expansion and the kinetic energy of depositing species, as well as facilitate reactive film growth [61]. Finally, the target-substrate distance affects the flux and kinetic energy of the species arriving at the substrate [78]. The duration of a laser pulse influences its interaction with a material, leading to distinct modification mechanisms and diverse applications. The variability of pulse lengths, specifically nanosecond, picosecond (ps), and femtosecond (fs) pulses, and their associated mechanisms, particularly in the context of PLD, MAPLE, and laser-induced periodic surface structures (LIPSS), play a critical role in determining material interaction dynamics, ablation efficiency, and surface morphology outcomes. The key to understanding these varied applications lies in how laser energy is absorbed and dissipated within the material, which is directly tied to the pulse duration relative to characteristic material relaxation times, such as the electron–phonon coupling time. Nanosecond (ns) pulses (10^−9^ s) cause thermal melting and vaporization, leading to significant heat-affected zones (HAZ) due to heat diffusion, making them suitable for PLD and MAPLE due to their cost-effectiveness and film adhesion benefits [62,79,80,81]. In contrast, ps (10^−12^ s) and fs (10^−15^ s) “ultrashort” pulses enable “cold” ablation by depositing energy rapidly into electrons, minimizing heat transfer to the lattice [82,83,84]. Material removal primarily occurs through non-thermal mechanisms like Coulomb explosion and phase explosion, resulting in negligible HAZ and high precision [84,85]. These ultrafast pulses are essential for applications requiring high precision, such as LIPSS [86,87,88], micromachining of sensitive materials [89], transparent material processing [90], LASIK eye surgery [91], and precise nanoparticle synthesis [92]. Ultimately, selecting the appropriate laser pulse duration is crucial for controlling laser–material interaction, thermal impact, and the quality of processed materials [93].

The choice of laser pulse duration is a critical parameter in laser applications (Table 3), dictating the fundamental mechanisms of laser–material interaction, the extent of thermal impact, and ultimately, the achievable precision and quality of the processed material. While ns pulses are well-suited for applications where controlled thermal processes are acceptable or desired (like PLD and MAPLE for thin film deposition), ultrafast fs and ps pulses enable “cold” ablation, opening doors to highly precise micromachining and novel surface structuring techniques like LIPSS, where minimizing thermal damage is paramount.

PLD offers several advantages for fabricating antimicrobial polymeric composite thin films, including its ability to deposit a wide range of complex materials, the potential for stoichiometric transfer, and the possibility of in situ doping and nanostructuring. However, challenges like particulate emission (droplets) from the target surface and achieving uniform deposition over large areas require careful optimization of process parameters and techniques such as target rotation and laser beam rastering [75].

Several key parameters influence the MAPLE process for polymeric composites. The laser wavelength should be strongly absorbed by the matrix solvent to ensure efficient evaporation; UV wavelengths are commonly used for organic matrices [61]. The laser fluence needs to be optimized to achieve efficient matrix evaporation and analyte transfer without causing sputtering or fragmentation of the analyte, with MAPLE typically operating at lower laser fluences compared to PLD [61].

The matrix solvent choice is crucial; it should have a high vapor pressure, be compatible with the analyte, and efficiently absorb the laser radiation [96]. Analyte concentration in the matrix should be low to minimize analyte–analyte interactions in the plume and promote the deposition of intact molecules or well-dispersed nanoparticles. Finally, the substrate temperature can influence the adhesion and morphology of the deposited film and can also aid in the evaporation of residual solvent [61].

MAPLE offers several advantages for antimicrobial polymeric composites. It ensures the preservation of analyte integrity, allowing the deposition of thermally labile organic molecules and fragile polymers with minimal decomposition. It enables controlled deposition of complex materials, including multi-component systems with polymers and nanoparticles, with relatively uniform distribution. Additionally, MAPLE often yields smooth and homogeneous films compared to PLD, especially for polymeric materials [75].

Despite its advantages, MAPLE has certain limitations. It typically has lower deposition rates compared to PLD due to the low concentration of the analyte in the target. Furthermore, target preparation can be challenging, especially for viscous polymer solutions or nanoparticle dispersions [61].

## 3. Composition of Laser-Deposited Antimicrobial Thin Films

The effectiveness and practical application of polymeric composite thin films produced by laser deposition for antimicrobial uses are primarily determined by their precise composition [97]. These films are typically designed as multi-component systems, carefully integrating a polymeric matrix, one or more antimicrobial agents, and often incorporating nanostructuring strategies to enhance performance [98]. The careful selection and synergistic combination of these components are vital for achieving the desired antimicrobial activity [99], biocompatibility [100], mechanical integrity [101], and overall functionality of the thin film [102]. This section will present key constituents of these advanced materials, exploring the characteristics and roles of the polymer matrices, the diverse range of incorporated antimicrobial agents, and the impact of nanostructuring on their antimicrobial properties.

To further elaborate on the characteristics of these composite films, Table 4 provides a summary of the advantages and disadvantages associated with key components, specifically polymer matrices and NPs, in the context of antimicrobial coatings.

### 3.1. Polymeric Composite Classification

A comprehensive understanding of polymeric composite thin films designed for antimicrobial applications and fabricated using laser deposition techniques necessitates a multifaceted classification. The polymeric matrix forms the continuous phase of the composite thin film, providing the necessary structural integrity, mechanical stability, and adhesion to the underlying substrate. The choice of polymer is paramount, as it influences not only the physical properties of the film but also its biocompatibility, permeability, and potential interactions with the incorporated antimicrobial agents. Several polymers have been explored as matrices for antimicrobial thin films deposited by laser techniques, each offering a unique set of advantages and considerations [109].

The classification is based on five key differentiating factors: the nature of the polymer matrix (synthetic or natural), the type of antimicrobial agent incorporated (metal/metal oxide nanoparticles, organic agents, or hybrid combinations), the laser deposition technique employed, the resulting film morphology and nanostructuring (nanoparticle-embedded, surface-nanostructured, layered, or porous), and the intended application area (biomedical, food packaging, water purification, or self-sterilizing surfaces). A single antimicrobial film can often be categorized under multiple aspects of this classification, highlighting these advanced materials’ complexity and tailored design.

#### Polymer Matrix Type (Synthetic vs. Natural)

**Synthetic polymers**

Poly(D,L-lactide) (PDLLA) has been successfully used as a matrix for incorporating antibiotics like gentamicin, with films fabricated via MAPLE showing strong antimicrobial activity against *S. aureus* [110]. Additional studies have shown that PDLLA can be deposited on 3D Bioglass^®^ scaffolds using MAPLE for potential osteochondral tissue engineering, forming hybrid graded materials [111].

A synthetic, water-soluble polymer, polyvinylpyrrolidone (PVP) has intrinsic adhesion and biocompatibility. Hybrid films of PVP and graphene-like materials (GL-PVP) deposited via MAPLE demonstrated good cytocompatibility across various human and murine cell lines and are proposed for flexible biomedical devices [112].

Functionalized PVA thin films (e.g., PVACOOH) have also been successfully fabricated using MAPLE. These materials support drug release and show desirable surface porosity and biocompatibility [113].

PEG is valued for its biocompatibility and antifouling properties. Thin films deposited by MAPLE using visible and infrared laser wavelengths retained the polymer’s native chemical structure, making them excellent candidates for in vivo applications and drug delivery coatings [114,115].

Polyvinylidene Fluoride (PVDF) is a piezoelectric and chemically stable synthetic polymer. MAPLE-deposited PVDF coatings support osteoblast survival and proliferation, with in vitro results indicating suitability for orthopedic and implant applications. Surface hydrophilicity and morphology were optimized without the need for post-deposition thermal treatment [116].

For example, one of the commonly employed polymers is poly(methyl methacrylate) (PMMA), also known as acrylic glass or Plexiglas. PMMA is a synthetic polymer known for its excellent optical transparency, good mechanical strength [117], biocompatibility, and ease of processing. Its amorphous nature allows for relatively uniform dispersion of incorporated nanoparticles, and its well-established use in biomedical applications makes it a suitable candidate for antimicrobial coatings on medical devices [118]. Studies utilizing laser deposition techniques, such as PLD, have demonstrated the feasibility of depositing thin films of PMMA with controlled thickness and morphology [119]. The ability to precisely ablate and deposit PMMA using lasers opens avenues for creating intricate patterns and localized antimicrobial functionalities on surfaces [119].

Another promising polymeric matrix is based on lignin, a complex biopolymer derived from plant biomass. Lignin itself exhibits inherent antimicrobial properties due to the presence of phenolic hydroxyl groups in its structure [120]. Grafting lignin with other polymers, such as polyaniline (PANI), can further enhance its properties, including improved electrical conductivity and processability [121]. PANI-grafted lignin offers a sustainable and potentially cost-effective alternative to purely synthetic polymers. The presence of polyaniline can also contribute to the antimicrobial activity through its redox properties and interaction with microbial cell membranes. Laser deposition of such bio-based polymer composites presents an environmentally friendly approach to fabricating antimicrobial surfaces with tailored functionalities [121].

In conclusion, the examples of PDLLA [111], PVP [112], functionalized PVA [113], PEG [114,115], PVDF [116], PMMA [118], and lignin-based composites [121] collectively demonstrate the immense versatility of laser deposition techniques, particularly MAPLE, for fabricating diverse polymeric thin films. These techniques enable the successful deposition of both synthetic and bio-based polymers, preserving their inherent properties such as biocompatibility, drug release capabilities, mechanical strength, and even intrinsic antimicrobial characteristics (as seen with lignin). The precise control offered by laser methods allows for tailoring film morphology, optimizing surface properties (e.g., hydrophilicity), and ensuring the functional integration of active components without degradation. This broad applicability across a range of polymer types, coupled with the ability to create complex and functionalized coatings, highlights the significant potential of laser deposition for developing advanced biomedical devices, tissue engineering scaffolds, and antimicrobial surfaces with highly tailored properties.

**Natural/biocompatible polymers**

Known for its inherent antimicrobial properties, chitosan was used in compositional gradients with hydroxyapatite using combinatorial MAPLE. The films demonstrated strong antimicrobial effects against *S. aureus* and *E. coli* and enhanced osseointegration potential [122].

Dextran, a natural polysaccharide, has been combined with iron oxide NPs to produce bioactive coatings via MAPLE. These films supported cell viability and showed potential as biomedical markers [123].

Natural compound usnic acid embedded in PLA–PVA microspheres via MAPLE was shown to inhibit *S. aureus* biofilm formation, with biocompatible coatings demonstrated on titanium substrates [96].

These were shown to improve hydrophilicity and mechanical properties while inhibiting biofilm formation. Such combinations are attractive for wound dressing and implant coatings [124].

The successful deposition of these diverse polymeric materials using laser techniques necessitates careful optimization of the laser parameters, including fluence, repetition rate, and wavelength, to ensure controlled ablation and film formation without significant thermal degradation of the polymer chains. The interaction between the laser plume and the substrate also plays a crucial role in determining the film’s adhesion and morphology [125]. The selection of the polymer matrix is not solely based on its processability via laser techniques and its structural properties.

Biocompatibility is a critical consideration, especially for applications involving direct contact with biological tissues or fluids. The polymer should ideally be non-toxic, non-immunogenic, and should not elicit adverse reactions from the host. Furthermore, the permeability of the polymer matrix can influence the release kinetics of the incorporated antimicrobial agents, affecting the longevity and efficacy of the antimicrobial activity. Therefore, a holistic approach considering the interplay between the polymer’s intrinsic properties, its processability via laser deposition, and the intended application is essential for the rational design of antimicrobial thin films [126].

Laser deposition techniques, in particular MAPLE, prove highly effective for fabricating advanced antimicrobial and bioactive films using diverse natural polymers like chitosan [122], dextran [123], and usnic acid composites [96]. These studies demonstrate significant antimicrobial efficacy, enhanced biocompatibility, and improved functional properties, making them promising for biomedical applications such as implants and wound dressings. Successful implementation hinges on meticulous optimization of laser parameters, careful consideration of polymer–substrate interactions, and a holistic design approach that prioritizes polymer biocompatibility and controlled release kinetics [126].

### 3.2. Type of Antimicrobial Agent

Another significant classification is based on the type of antimicrobial agent. The incorporation of specific antimicrobial agents within the polymeric matrix is the primary strategy for imparting antimicrobial functionality to the thin films. A wide range of substances, exhibiting diverse mechanisms of action against various microorganisms, have been successfully integrated into laser-deposited polymeric films. These agents can be broadly categorized into inorganic NPs, organic molecules, and even biomacromolecules.

#### 3.2.1. Metal and Metal Oxide NPs

Antimicrobial polymeric thin films have been developed using a variety of agents, broadly categorized into metal/metal oxide-based, organic, and hybrid composites.

**Figure 4:**
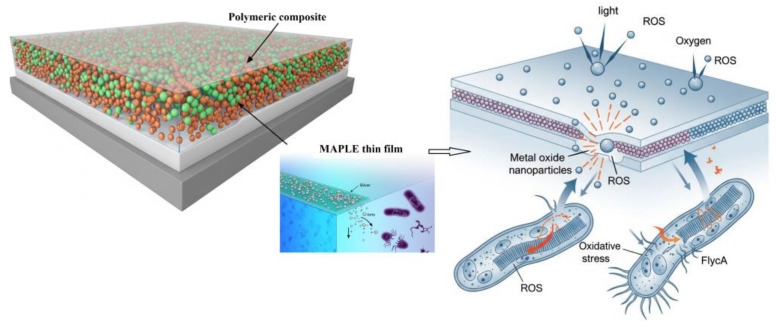
Proposed mechanism of antibacterial activity of the developed materials. The left panel shows a polymeric composite layer deposited on a substrate, with an underlying MAPLE thin film. The right panel illustrates how light interacts with metal oxide NPs within the film, leading to the generation of ROS from oxygen. These ROS then induce oxidative stress in bacteria, affecting their viability and potentially involving mechanisms like FlycA.

Among the metal-based agents, silver NPs (Ag NPs) are well-known for their broad-spectrum and potent antimicrobial activity and have been successfully incorporated into polymer matrices via laser-based methods like MAPLE and PLD [127]. Mechanisms include the release of Ag^+^ ions, which can disrupt microbial cell membranes, interfere with metabolic processes, and damage DNA [128]. The high surface area-to-volume ratio of NPs enhances their interaction with microbial cells, leading to potent antimicrobial effects even at low concentrations [129] (Figure 4).

Laser tailoring of Ag NPs through shape- and size-specific laser-induced surface diffusion can significantly narrow their size distribution, directly influencing antimicrobial activity and optical properties [130].

In situ NP formation in polymer matrices using femtosecond laser pulses enables localized synthesis and structural control of Ag NPs, which allows tuning of particle characteristics that affect efficacy and biocompatibility [131].

Dual pulsed laser deposition (DPLD) enables sequential Ag NP deposition over calcium phosphate layers, achieving uniform Ag NP size control and modulating antimicrobial activity while supporting osteoblast viability [132].

DPLD is an advanced PLD technique that uses two independent laser pulses or beams to ablate material and interact with the plasma plume, offering significantly enhanced control and flexibility over conventional single-pulse PLD [133]. This can involve sequential ablation from a single target to improve film density, adhesion, crystallinity, and purity by reducing macroscopic particles, or simultaneous/sequential ablation from two different targets to create composite or alloy films [132]. DPLD’s numerous advantages include superior manipulation of the plasma plume for improved film quality (density, crystallinity, smoothness, adherence), wider material versatility for complex alloys and heterostructures, and cleaner deposits [134]. These capabilities make DPLD invaluable for fabricating highly specialized thin films for advanced applications, including complex oxides, quantum materials, functional ceramics, and biomedical coatings, by enabling precise control over stoichiometry and functional properties [133,135].

PLD allows controlled Ag NP growth with narrow size distributions, affecting NPs surface coverage, size, and plasmonic response, all critical for biological interactions [136].

Laser-assisted polymer nanocomposite deposition (plasma-based) enables embedding Ag NPs within a plasma polymer matrix, where NP concentration and matrix thickness control release rates and bioactivity [137].

Beyond Ag NPs, other metallic NPs, such as copper NPs (Cu NPs) and gold NPs (Au NPs), have also demonstrated promising antimicrobial properties and are being explored for incorporation into laser-deposited polymeric films.

CuNPs are known for their broad-spectrum antimicrobial activity, and their inclusion in polymeric coatings can be effective against both Gram-positive and Gram-negative bacteria. However, their oxidation state and potential cytotoxicity require controlled stabilization. For example, multiple studies demonstrate CuNPs’ antimicrobial potential in surface coatings, including laser-deposited composites [138].

Au NPs are generally more biocompatible and exhibit size- and shape-dependent antimicrobial properties. Functionalization of Au NPs allows for enhanced interactions with microbial membranes, contributing to their antibacterial performance [139]. Their use in MAPLE and PLD-deposited polymeric films has also been explored to achieve controlled release and improved bioactivity.

Laser deposition techniques have demonstrated the ability to precisely control nanoparticle distribution, shape, and size within a polymer matrix, which in turn affects both antimicrobial efficacy and cytotoxicity. A study by Poletti et al. demonstrated the synthesis of gold “nanocorals” via laser irradiation, highlighting their excellent biocompatibility and suitability for incorporation into polymeric matrices, showing no cytotoxic effects even at high doses [140]. Photocatalytic metal oxides like titanium dioxide (TiO_2_) and zinc oxide (ZnO) are frequently used for their light-activated antimicrobial effects and have been effectively integrated into composite films for enhanced performance [141].

Organic antimicrobial agents embedded in polymeric films include antibiotics like gentamicin, which have been shown to retain their activity when delivered via polymer matrices, especially in thin film formats designed for biomedical applications [142]. These films can also incorporate antifungal or antiviral compounds for broader-spectrum efficacy.

Hybrid antimicrobial composites, which combine metals, metal oxides, and organic agents, are gaining attention for their synergistic effects. For instance, Ag/ZnO hybrid films have demonstrated enhanced antibacterial activity compared to single-agent films, highlighting the benefit of combining photocatalytic and ion-release mechanisms [143].

Metal oxides, such as TiO_2_ and ZnO, are another class of inorganic antimicrobial agents that can be incorporated into polymeric thin films deposited by laser techniques. TiO_2_ NPs, particularly in their photocatalytic form, can generate reactive oxygen species (ROS) upon exposure to UV or visible light, which can effectively kill microorganisms by oxidizing their cellular components. Titanium dioxide exists in several crystalline forms, or polymorphs, the most common and relevant for photocatalysis being anatase, rutile, and brookite. While all three can exhibit some level of photocatalytic activity, their efficiencies vary significantly due to differences in their crystal structure, electronic band structure (specifically band gap energy and electron–hole recombination rates), surface area, and morphology. Among these, anatase is generally considered the most active and preferred photocatalytic form of TiO2 for many applications, including antimicrobial ones [144]. ZnO NPs also exhibit antimicrobial activity through various mechanisms, including the release of zinc ions and the generation of ROS. Laser deposition offers a versatile method for incorporating these metal oxide NPs into polymeric matrices, potentially leading to self-sterilizing surfaces that are activated by light [145].

To conclude, laser deposition techniques are revolutionizing the development of antimicrobial coatings by providing unprecedented control over NPs characteristics, including size, shape, distribution, and surface coverage, as well as their integration into polymer matrices. This remarkable precision is needed for optimizing antimicrobial efficacy, enhancing biocompatibility, and enabling controlled release mechanisms, thereby addressing critical requirements for advanced biomedical and surface applications [140].

Among various antimicrobial agents, Ag NPs continue to be a leading choice, largely due to the exceptional control afforded by laser-based deposition methods. These techniques allow for the precise tuning of Ag NP size distribution and surface properties, which is crucial for maximizing their antimicrobial effect while simultaneously mitigating potential adverse biological interactions [137].

Beyond Ag, laser deposition facilitates the effective incorporation of a diverse array of other antimicrobial agents. Cu NPs, known for their broad-spectrum activity, can be integrated, though careful stabilization is necessary [138]. Au NPs are notable for their superior biocompatibility and highly tunable antimicrobial properties, with laser-induced synthesis enabling unique structures like “nanocorals” that show significant promise [140]. Furthermore, photocatalytic metal oxides such as titanium dioxide and zinc oxide are gaining importance. Their light-activated, self-sterilizing capabilities, made possible by laser deposition, open new avenues for developing durable and renewable antimicrobial surfaces [145].

A significant advancement in this field is the creation of hybrid antimicrobial composites, which combine different agents to leverage synergistic effects. For instance, the combination of ion release and photocatalysis can lead to highly potent, multi-mechanistic antimicrobial surfaces, pushing the boundaries of effectiveness beyond single-agent systems [143]. Moreover, the capacity of laser-deposited polymer films to embed organic antimicrobial agents, such as antibiotics, while preserving their activity, highlights their immense versatility for localized and sustained drug delivery in a variety of biomedical applications.

#### 3.2.2. Organic Antimicrobial Molecules

In addition to inorganic NPs, organic antimicrobial molecules can also be incorporated into laser-deposited polymeric thin films.

The choice of the antimicrobial agent depends on several factors, including the target microorganisms, the desired level and duration of antimicrobial activity, potential toxicity to mammalian cells, and compatibility with the laser deposition process and the chosen polymer matrix. Often, a combination of different antimicrobial agents with complementary mechanisms of action can lead to synergistic effects and a broader spectrum of antimicrobial activity, mitigating the risk of resistance development. Laser deposition techniques offer the flexibility to create such multi-agent composite films with precise control over the spatial distribution and concentration of each component [146].

The integration of organic antimicrobial agents into polymeric composite thin films has gained significant attention for biomedical and environmental applications, particularly when combined with precision laser deposition techniques such as MAPLE. These techniques allow for the gentle transfer of delicate organic molecules onto substrates while preserving their chemical structure and biological function.

Table 5 categorizes commonly used organic antimicrobial agents, highlighting their role in polymeric thin films fabricated by MAPLE or similar laser techniques, and includes real, peer-reviewed references for each example.

One of the most studied organic antimicrobial agents in laser-deposited films is gentamicin, an aminoglycoside antibiotic. Thin films composed of poly(1,3-bis-(p-carboxyphenoxy propane)-co-sebacic anhydride) embedded with gentamicin were fabricated using MAPLE. These coatings exhibited strong antibacterial activity against *E. coli* and *S aureus*, and showed uniform morphology with minimal degradation of the antibiotic during deposition [147].

Another example involves the use of essential oils, such as cypress oil, known for their antimicrobial properties. Stefan et al. developed a hybrid polymeric coating by embedding Fe_3_O_4_ NPs coated with cypress essential oil into a PLGA matrix. The resulting films, deposited by MAPLE, exhibited antimicrobial efficacy against *S. aureus*, *E. coli*, and *C. albicans*, along with high biocompatibility for osteoblast-like cells [151].

Organic antimicrobial films can also include polymer-drug composites made from biodegradable polymers like PDLLA, blended with antibiotics or antifungal agents. Cristescu et al. demonstrated that such films maintain their antimicrobial effectiveness when transferred via MAPLE, suggesting their utility for medical device coatings [110].

Moreover, terpenoid-based polymers, derived from natural compounds like terpinen-4-ol (found in tea tree oil), have been polymerized into antimicrobial coatings via plasma-enhanced methods. These organic thin films significantly inhibited *Pseudomonas aeruginosa* biofilm formation, and their efficacy depended on the preservation of active functional groups during the deposition process [152].

One significant development includes the fabrication of composite coatings containing ciprofloxacin, a broad-spectrum antibiotic, incorporated into a bioglass–polymer matrix for titanium implants. The dual-layer coatings, polymer base followed by bioglass–ciprofloxacin layer, showed notable antibacterial effects against *S. aureus*, *E. faecalis*, *E. coli*, and *P. aeruginosa*, along with biocompatibility for osteoblast cells [100].

Isoflavonoid-antibiotic combinations have also been explored for their anti-adhesive properties. Grumezescu et al. fabricated thin films composed of polyvinylpyrrolidone, antibiotics, and isoflavonoids via MAPLE. These coatings resisted microbial colonization by *S. aureus* and *P. aeruginosa* and demonstrated excellent cytocompatibility with endothelial cells, making them promising candidates for anti-biofilm biomedical surfaces [156].

For advanced drug delivery and stimuli-responsive systems, gentamicin-loaded Fe_3_O_4_ NPs embedded in polyaniline-grafted lignin (PANI-LIG) matrices have been deposited onto titanium via MAPLE. These coatings allowed for magnetically and electrically controlled antibiotic release and showed broad-spectrum antimicrobial action while supporting osteoblast proliferation [121].

Moreover, combinatorial MAPLE has enabled the fabrication of thin films with gradient compositions of antimicrobial biopolymers. These smart coatings, featuring sulfated levan and quaternized chitosan, displayed antimicrobial effects against both *E. coli* and *S. aureus* and varied bioactivity along the compositional gradient. This approach offers a promising route for tunable, anti-infective biomaterials [157].

MAPLE has proven particularly valuable due to its ability to deposit uniform, functional organic coatings without damaging labile compounds, as initially demonstrated for a variety of organic materials, including polysiloxanes and carbohydrates [158].

MAPLE is particularly well-suited for the deposition of delicate organic molecules, as it involves dissolving the molecule in a volatile solvent matrix, which is then laser-ablated, transferring the intact molecule onto the substrate. This technique minimizes thermal degradation of the organic antimicrobial agent during the deposition process. The release kinetics of these organic molecules from the polymer matrix can be controlled by the polymer’s properties and the morphology of the film [159].

In conclusion, laser deposition techniques, particularly MAPLE, provide a highly versatile platform for creating advanced antimicrobial coatings by integrating both inorganic NPs and diverse organic agents into polymeric thin films [146]. This precision is vital for biomedical and environmental uses, allowing gentle transfer of delicate molecules while preserving their function. Careful agent selection, considering target organisms, activity, toxicity, and compatibility, is crucial. Furthermore, laser deposition’s flexibility enables multi-agent composites with precise control over component distribution and concentration, resulting in synergistic effects, broader antimicrobial spectra, and reduced resistance [158].

Significantly, organic antimicrobial agents, including various antibiotics (e.g., gentamicin [147], ciprofloxacin [100]) and natural compounds (essential oils [151], propolis [153]), have been successfully integrated into polymer matrices via MAPLE. These films consistently exhibit strong antibacterial activity against common pathogens while maintaining biocompatibility. A key advantage of laser deposition for organics is its ability to minimize thermal degradation by facilitating intact molecule transfer. These techniques also offer precise control over film morphology and release kinetics, enabling tunable drug delivery [159]. Consequently, emerging developments, such as smart, stimuli-responsive, and gradient-composition coatings, underscore the advanced control offered by laser methods for highly customized and effective antimicrobial surfaces.

## 4. Antimicrobial Efficacy of Laser-Deposited Polymeric Composite Thin Films

### 4.1. Antimicrobial Efficacy Testing

A major challenge in the comparative assessment of laser-deposited antimicrobial coatings arises from the widespread variability in antimicrobial testing methodologies. Table 6 summarizes these techniques. The diversity in assay types, bacterial strains, inoculum concentrations, and incubation conditions significantly hampers reproducibility and obstructs efforts to draw meaningful comparisons across studies.

Among the most commonly used evaluation techniques is the ZOI assay. It is a rapid and visually intuitive method; however, it primarily measures the diffusion of antimicrobial agents into the surrounding medium. Consequently, ZOI is poorly suited to assessing non-leaching, contact-killing surfaces that rely on direct bacterial interaction rather than diffusion-based mechanisms [160].

The CFU counting method remains a gold standard for quantitative analysis of bacterial viability on surfaces. Nevertheless, it is labor-intensive and may underestimate viable populations due to the exclusion of viable but non-culturable (VBNC) cells. Despite its quantitative rigor, CFU counting is highly sensitive to variables such as bacterial growth phase, nutrient medium, and incubation time [161].

To complement culture-based methods, fluorescence-based live/dead staining (e.g., SYTO9/propidium iodide) is increasingly used to visualize membrane-compromised bacteria directly on test surfaces. This method is particularly useful for assessing nanopatterned and contact-active materials. However, it requires specialized imaging systems and may yield false positives due to transient changes in membrane permeability, which can overestimate antimicrobial effects.

Metabolic assays, such as MTT or resazurin reduction tests, offer rapid, high-throughput screening by measuring metabolic activity as a proxy for viability. Yet, these assays are often affected by material-specific interactions with reagents, leading to potential inaccuracies. Similarly, biofilm quantification methods like the crystal violet assay are valuable for evaluating long-term bacterial colonization but lack the ability to differentiate between live and dead cells. Their poor sensitivity further complicates interpretation in thin film systems [162].

Despite the availability of international standards such as ISO 22196 and ASTM E2149, inter-laboratory variability persists. Factors such as bacterial strain variation, growth phase (stationary vs. exponential), inoculum concentration, and environmental conditions (temperature, humidity, media composition) significantly influence assay outcomes. Round-robin trials have confirmed inconsistent results, particularly for materials with moderate antimicrobial performance [161].

In response to limitations of traditional methods, ISO 7581:2023 has introduced a “dry test” model intended to mimic real-world conditions more accurately. While this approach improves ecological validity, it may reduce reproducibility due to greater environmental variability [163]. Additional studies advocate for combining ISO 22196 or JIS Z 2801 with zone of inhibition methods to discern leaching from surface-bound effects [162].

### 4.2. Mechanisms of Antimicrobial Action

The ultimate utility of polymeric composite thin films deposited by laser techniques lies in their ability to effectively inhibit or eradicate microbial growth. This section is dedicated to the antimicrobial efficacy of these innovative materials, exploring the mechanisms of action, the methodologies employed for evaluating their performance, and the key factors that influence their effectiveness against a broad spectrum of pathogens. Furthermore, we will discuss the current state of research concerning their activity against specific microorganisms and highlight the challenges and future directions in optimizing their long-term antimicrobial performance for real-world applications.

**Figure 5:**
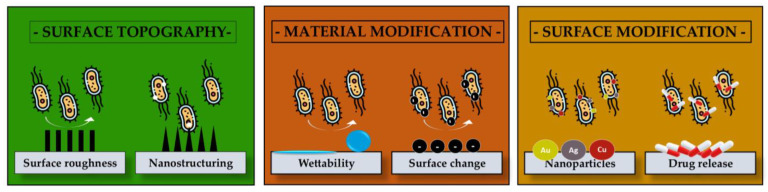
Illustration of surface engineering strategies for antimicrobial coatings: green square—surface topography modulates bacterial adhesion through surface roughness and nanostructuring; red square—material modification alters wettability and surface charge to disrupt microbial attachment; tan square—surface functionalization enhances antimicrobial activity via NP interaction (e.g., Au, Ag, Cu) and controlled drug release.

Laser-deposited polymeric composite thin films exert their antimicrobial effects through various mechanisms, often acting synergistically depending on the incorporated antimicrobial agents and the physicochemical properties of the film surface (Figure 5).

Two primary modes of action are commonly observed:

(i) The release of active antimicrobial agents into the surrounding environment

One of the primary mechanisms through which laser-deposited polymeric composite thin films exert their antimicrobial activity is by the controlled release of embedded bioactive agents. These agents, which can include antibiotics, essential oils, NPs, or antifungals, are incorporated into the film matrix and gradually diffuse into the surrounding environment to inhibit microbial growth. Laser-based deposition techniques, such as MAPLE and PLD, enable precise control over film composition and structure, allowing for tailored drug release profiles. The released agent then interacts with microbial cells in the surrounding environment, inhibiting their growth or causing their death. The kinetics and extent of this release are critically dependent on factors such as the type and concentration of the antimicrobial agent, its dispersion within the polymer matrix, the porosity and permeability of the film, and the surrounding medium (e.g., aqueous solution, biological fluid) [164].

Metallic NPs, particularly AgNPs, frequently used in these composite films, exert their antimicrobial action partly through the release of silver ions (Ag+). These ions are known to interact with various cellular components, including the cell membrane, proteins, and DNA, leading to disrupted cellular functions and ultimately cell death. The rate of silver ion release from the polymer matrix is influenced by the size and surface area of the AgNPs, their interaction with the polymer, and environmental conditions like pH and the presence of certain ions. Laser deposition techniques can influence the size and dispersion of AgNPs within the polymer, thereby indirectly controlling the silver ion release profile [165]. Metal oxide NPs, such as TiO_2_ and ZnO, can also exhibit antimicrobial activity through the release of metal ions (e.g., Zn^2+^ from ZnO). Additionally, TiO_2_ NPs, upon activation with UV or visible light, can generate reactive oxygen species (ROS), such as hydroxyl radicals and superoxide anions [166]. These ROS are potent oxidizing agents that can damage microbial cell membranes, proteins, and DNA, leading to cell inactivation [167]. The laser deposition process can influence the crystallinity and surface area of the metal oxide nanoparticles, which in turn affects their photocatalytic activity and ion release [168].

In another polymer-integrated system, Ag-PEI-PLA composite films demonstrated dual action by releasing silver ions and directly contacting and disrupting bacterial membranes, particularly relevant in the prevention of denture stomatitis [169].

Similarly, organic antimicrobial agents, such as antibiotics (e.g., ciprofloxacin) incorporated into the polymer matrix, exert their antimicrobial effects upon their release from the film. The polymer matrix acts as a reservoir, providing a sustained release of the drug to the surrounding environment, leading to localized antimicrobial activity. The release rate is governed by the diffusion of the drug through the polymer network, influenced by the polymer’s density, cross-linking, and swelling behavior [170].

**Figure 6:**
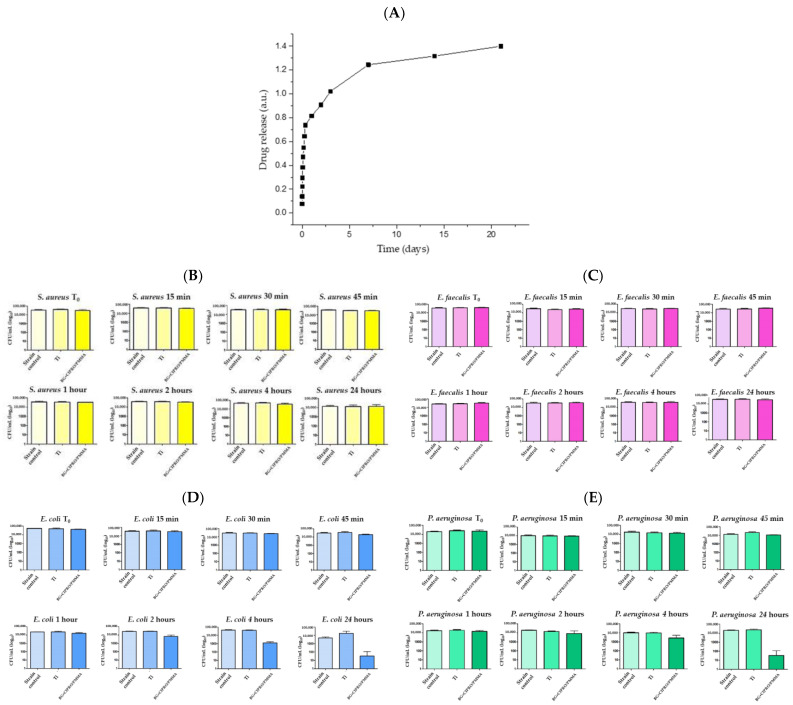
(**A**) Drug release as a function of time from BG+CIPRO/PMMA coatings; (**B**) evaluation of *S. aureus* viability in the presence of bare Ti and BG+CIPRO/PMMA samples; (**C**) evaluation of *E. faecalis* viability in the presence of bare Ti and BG+CIPRO/PMMA samples; (**D**) evaluation of *E. coli* viability in the presence of bare Ti and BG+CIPRO/PMMA samples and (**E**) evaluation of *P. aeruginosa* viability in the presence of bare Ti and BG+CIPRO/PMMA samples [100].

For example, Neguț et al. developed MAPLE-deposited ciprofloxacin-bioglass-polymethylmethacrylate (BG+CIPRO/PMMA) composite coatings that released ciprofloxacin over time, showing broad-spectrum antibacterial activity against *S. aureus*, *E. faecalis*, *E. coli*, and *P. aeruginosa* (Figure 6) [100].

In another study, Stefan et al. created PLGA-based films incorporating Fe_3_O_4_ nanoparticles and essential cypress oil, deposited by MAPLE. These films demonstrated antimicrobial effects through external field-triggered release of essential oil, effectively targeting *S. aureus*, *E. coli*, and *C. albicans* [151].

Visan et al. reported a nanostructured coating composed of polyaniline-grafted lignin (PANI-LIG) embedded with gentamicin-functionalized Fe_3_O_4_, which enabled a stimuli-responsive drug release under magnetic or electric fields, effectively controlling bacterial populations such as *E. coli*, *S. aureus*, and *C. albicans* [121].

Similarly, Neguț et al. showed that polyvinylpyrrolidone (PVP) thin films embedded with voriconazole and flavonoids could release active antifungal agents, maintaining antifungal efficacy against *C. albicans* and *C. parapsilosis* after MAPLE deposition [74].

**Figure 7:**
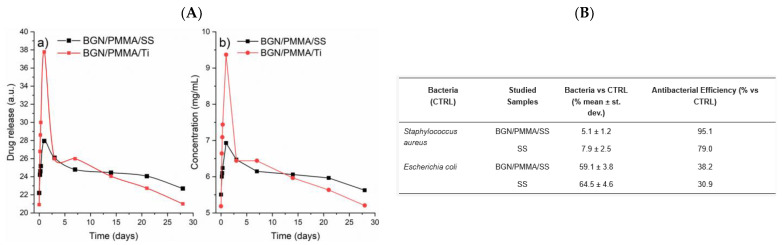
(**A**) Drug release as a function of time for BGN/PMMA/SS (black curve) and BGN/PMMA/Ti (red curve): (**a**) peak absorbance intensity function of time and (**b**) concentration function of time; (**B**) figure depicting the antibacterial activity of the samples against *S. aureus* and *E. coli* [154].

In another work, Neguț et al. reported a multifunctional nanostructured coating composed of BG, PMMA, and neem extract (NEEM), deposited by MAPLE, which demonstrated significant antimicrobial efficacy against *E. coli*, *S. aureus*, and *C. albicans* (Figure 7). The coating leveraged both the bioactivity of BG and the phytochemical properties of NEEM, achieving sustained microbial inhibition and improved surface compatibility for biomedical application [154].

In a similar study, Floroian et al. reported a nanostructured MAPLE-deposited thin film composed of bioactive glass blended with turmeric and *Ocimum sanctum* (holy basil) extracts, deposited onto stainless-steel implant surfaces. This coating provided enhanced bioactivity—evidenced by apatite layer formation in simulated body fluid—and delivered sustained antimicrobial effects, prominently inhibiting *E. coli*, *S. aureus*, and *C. albicans* through gradual phytochemical release and contact killing, while also serving as a barrier to metal ion release [171].

Elabbasy et al. employed PLD to fabricate silver nanoparticle-loaded CMC-PVA composite films for wound dressings. The antimicrobial effect was attributed to sustained Ag ion release, which efficiently inhibited *E. coli*, *S. aureus*, *P. aeruginosa*, and *Micrococcus luteus* [172].

Another work by Popescu-Pelin et al. used PLD to create fish bone-derived bi-phasic calcium phosphate coatings, which demonstrated antimicrobial effects via the slow release of trace elements (Na, Mg, Si, S), contributing to reduced biofilm formation by *E. coli* [173].

Porphyrin-functionalized coatings deposited by PLD and MAPLE also released antimicrobial agents capable of preventing biofilm formation and corrosion on metallic surfaces, offering dual antibacterial and protective functionality [174].

A novel combinatorial MAPLE method by Sopronyi et al. synthesized carbon–cobalt oxide nanocomposite films, where embedded CoO NPs exhibited antimicrobial properties through their controlled release from a carbon matrix, modulated by NPs distribution gradients [175].

Cocean et al. explored the use of natural chitosan thin films processed by high-power laser techniques, which released antimicrobial biopolymers effective for wound care and filtration applications [176].

Another application used hydroxypropyl methylcellulose (HPMC) and ethyl cellulose (EC) polymer blends embedded with captopril to fabricate drug-eluting transdermal patches. The laser-engineered thin films demonstrated adjustable drug release profiles depending on polymer ratios, suggesting similar feasibility for controlled antimicrobial delivery [177].

For the antifungal domain, Neguț et al. developed composite films of amphotericin B and resveratrol, showing synergistic antifungal activity from their co-release from a MAPLE-deposited matrix [74].

Lastly, hybrid composites of graphene oxide with laser-deposited silk fibroin showed promise for ion-mediated antimicrobial activity, leveraging both Ag release and polymer degradation for biofilm control [178].

In conclusion, laser-deposited polymeric composite thin films primarily exert their antimicrobial activity through the controlled release of embedded bioactive agents, including antibiotics, essential oils, and metal/metal oxide NPs [179]. These advanced techniques enable precise control over film composition, structure, and thus, the kinetics and extent of agent release [158]. This mechanism allows for sustained antimicrobial action against a broad spectrum of microorganisms by various means, such as ion release (Ag^+^, Zn^2+^) [180,181], reactive oxygen species generation (TiO_2_) [167], or direct drug diffusion [100]. The versatility of this approach extends to diverse polymer matrices and active agents, showcasing significant potential for tailored and effective antimicrobial surface applications.

(ii) Direct contact interaction with microbial cells, leading to their disruption.

Laser-deposited polymeric composite thin films are not solely dependent on drug release for antimicrobial action. A key alternative mechanism is contact killing, wherein microbial cells are inactivated through direct interaction with the surface of the coating [182]. These effects are influenced by the physicochemical properties of the films, such as surface roughness, hydrophilicity, electroactivity, and the presence of functional nanomaterials, and are critical in preventing biofilm formation and initial microbial colonization.

Here is how contact is made and how key environmental and material factors influence this mechanism. Surface contact and membrane disruption is one of them.

Bacterial membranes are negatively charged and composed of a high proportion of lipids and proteins. When they come into contact with positively charged or nanostructured surfaces, several effects can happen:

- Electrostatic interactions: Cationic polymers or functional groups (e.g., quaternary ammonium, polyaniline) can interact electrostatically with bacterial membranes, disrupting membrane integrity [183].
- Hydrophobic interactions: Hydrophobic patches on the film can destabilize bacterial membranes, especially for Gram-negative bacteria with outer lipid membranes [184].
- Mechanical puncture or deformation: Nanostructures, such as sharp nanopillars or ridges, can physically rupture membranes, similar to a “bed-of-nails” effect [182].

Also, the antibacterial performance of biomaterial surfaces is critically influenced by several physicochemical and environmental factors that govern microbial adhesion, survival, and biofilm formation.

By tuning properties such as surface wettability, pH responsiveness, thermal behavior, and nanoscale architecture, researchers can enhance the inherent antimicrobial efficacy of coatings and implants without relying solely on chemical agents. Each of these parameters affects the interaction between bacterial cells and material surfaces in distinct yet often complementary ways. The following sections outline the roles of key factors, including hydrophobicity/hydrophilicity, pH, temperature, and surface nanostructuring, with relevant examples from the recent literature highlighting their contributions to surface-mediated antimicrobial activity.

Understanding the mechanisms by which these factors operate is essential for the rational design of next-generation antibacterial coatings. Table 7 summarizes the significant factors involved, their underlying mechanisms, and their specific influence on contact-based bacterial killing.

### 4.3. Influence of Key Environmental Factors

a. Hydrophobicity/hydrophilicity

Hydrophilic surfaces can reduce bacterial adhesion by limiting protein and lipid adsorption, which are necessary for biofilm formation [186]. Hydrophilic surfaces are crucial for reducing bacterial adhesion on biomaterials by minimizing the initial adsorption of proteins and lipids [190]. For proteins, hydrophilic materials form a stable hydration layer that energetically repels protein adsorption, requiring significant energy for displacement, and can also induce steric repulsion, particularly with polymer modifications like PEG [191]. For lipids, highly hydrophilic surfaces diminish the hydrophobic interactions necessary for stable monolayer formation, leading to significantly reduced lipid adsorption [192]. This combined ability to resist both protein and lipid initial adsorption is fundamental for designing biocompatible surfaces that effectively prevent early biofilm formation and mitigate host immune responses [193]. Conversely, moderately hydrophobic surfaces can increase contact with bacterial lipid membranes, promoting membrane rupture [185]. Laser deposition can tailor the surface energy and wettability of films by controlling polymer composition, nanoparticle distribution, and surface morphology. Preventing initial microbial adhesion can significantly reduce the risk of subsequent colonization and infection. The interplay between antimicrobial agent release and direct contact killing can lead to a synergistic enhancement of overall antimicrobial efficacy [194].

**Figure 8:**
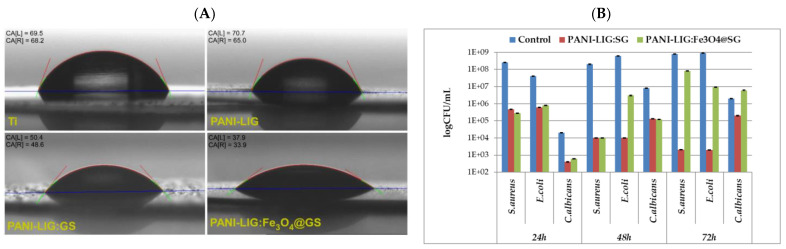
(**A**) Water droplets on bare Ti and Ti with PANI-LIG-based coatings deposited by MAPLE. Here, CA(L) represents the contact angle measured from the left side of the droplet, and CA(R) signifies the contact angle measured from the right side; (**B**) colony-forming unit (CFU)/mL values for *S. aureus*, *E. coli*, and *C. albicans* biofilms after 24, 48, and 72 h of incubation in the presence of control (bare substrates) and MAPLE nano-modified coatings [121].

PANI-LIG composites loaded with gentamicin-functionalized Fe_3_O_4_ NPs, deposited by MAPLE, presented significantly reduced contact angles (enhanced hydrophilicity) (Figure 8A) and electroactivity. These surface properties facilitated bacterial membrane destabilization upon contact, particularly against *E. coli*, *S. aureus*, and *C. albicans* [121].

**Figure 9:**
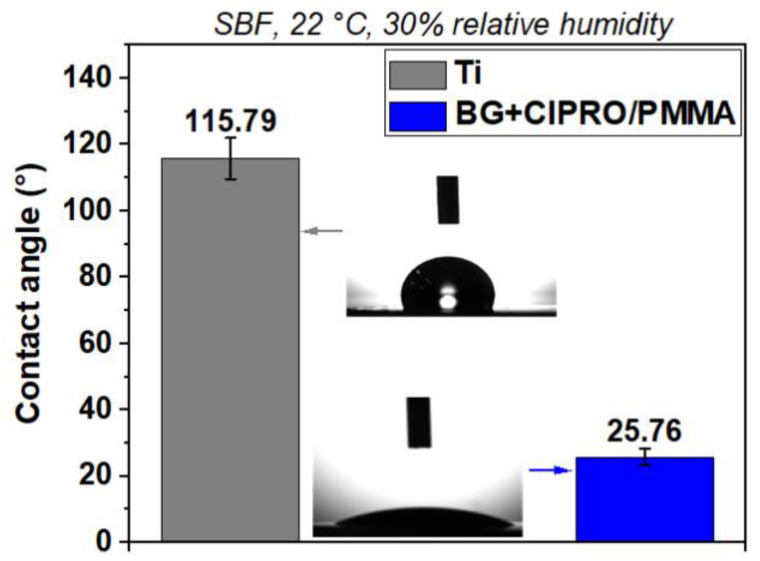
Contact angle measurements on bare Ti and Ti substrate coated with BG+CIPRO/PMMA [100].

In a similar study, Negut et al. showed that after applying a BG+CIPRO/PMMA coating on the substrate, the contact angle was decreased significantly to 25.76°; this effect can also facilitate bacterial membrane destabilization upon contact (Figure 9) [100].

b. pH

pH affects surface charge (via protonation/deprotonation of polymers) and bacterial membrane fluidity [187]. Cationic polymers like chitosan are more active in acidic pH, where they are protonated and can bind tightly to bacterial membranes [195]. Chitosan-based coatings deposited via MAPLE exhibit enhanced activity at pH ~5.5–6.0 due to stronger charge–membrane interactions [176].

The antimicrobial effectiveness of polymeric thin films fabricated via MAPLE or other laser techniques is profoundly influenced by ambient pH. pH affects the solubility, ionization, and release rate of embedded bioactive agents such as antibiotics, essential oils, and metal NPs [196].

BG+CIPRO/PMMA composite coatings deposited by MAPLE on Ti substrates exhibited potent antibacterial activity against *S. aureus*, *E. coli*, and *P. aeruginosa*. Ciprofloxacin’s activity is pH-dependent, with enhanced solubility and bacterial uptake at mildly acidic pH, making these coatings especially relevant for early-stage wound infections where local acidosis is common [100].

Similarly, hybrid MAPLE coatings of cypress essential oil, Fe_3_O_4_ NPs, and PLGA were found to be effective against *S. aureus*, *E. coli*, and *C. albicans*. Since essential oils are subject to pH-induced hydrolysis, their antimicrobial efficacy is sensitive to ambient pH, with acidic conditions promoting more effective release from the polymer matrix [151].

In another study, gradient thin films composed of sulfated levan and quaternized chitosan were fabricated using a combinatorial MAPLE approach. These coatings showed compositionally driven, pH-sensitive, anti-adhesion, and anti-biofilm properties, particularly effective against both *E. coli* and *S. aureus* [157].

PVP-based films loaded with voriconazole and flavonoids were also deposited using MAPLE. The antifungal activity of these coatings was shown to be modulated by pH, as voriconazole’s solubility and the ionization of flavonoids such as resveratrol are pKa-dependent, with optimal efficacy observed at slightly acidic to neutral pH, which mirrors mucosal and wound environments [74].

Another example includes hybrid films of zinc phthalocyanine (ZnPc) and ZnO NPs nanoparticles. ZnO is known to generate reactive oxygen species (ROS) and release Zn^2+^ ions more efficiently under acidic conditions, which enhances its antimicrobial properties. These films showed both photodynamic and chemical antibacterial effects that are inherently pH-sensitive [197].

Additionally, isoflavonoid–antibiotic thin films developed by MAPLE demonstrated resistance to microbial colonization. Although tested primarily at physiological pH, isoflavonoids (like genistein or daidzein) are known to undergo ionization shifts that affect their interaction with microbial cell walls depending on local pH, suggesting potential tuning of efficacy based on wound type or infection stage [156].

c. Temperature

Higher temperatures increase bacterial membrane fluidity and may enhance susceptibility to mechanical damage from surface contact. Temperature can also influence surface energy and polymer conformations, altering bacterial adhesion [198].

Ti-V-O oxide coatings laser-textured for dental implants showed increased antibacterial performance at body temperature due to better surface contact and oxidative interactions [199].

Paneysar et al. demonstrated that thermal-responsive AgNP release is tuned to infection-related temperature changes. Pullulan–pNIPAM–AgNP films released Ag in a temperature-triggered manner (above 37 °C). These films demonstrated antibacterial activity against *S. aureus* and *E. coli* and retained biocompatibility [200].

d. Nanostructures and surface topography

Nanopatterned surfaces can mechanically stress or puncture bacterial cells, even without chemical agents. Specific arrangements like nanopillars, ridges, or nanodomes mimic natural bactericidal surfaces (e.g., cicada wings) [201]. Sharp nanoscale features can physically penetrate or deform the microbial cell wall and membrane upon contact, leading to leakage of intracellular contents and cell lysis. The density, height, and sharpness of these nanostructures, which can be controlled to some extent by laser deposition parameters and specific nanomaterial incorporation, play a crucial role in this contact-killing mechanism [202].

The mechanisms of enhanced antimicrobial activity via nanostructuring are as follows:

- Increased surface area at the nanoscale facilitates stronger contact interactions between the film and microbial cells, improving contact-killing efficiency and diffusion-controlled drug release [203].
- Nanostructured surfaces increase the likelihood of direct contact between bacteria and bioactive agents (e.g., metal NPs or antibiotics). This proximity improves membrane disruption, ion release, and interaction with microbial cell walls. Greater surface area allows better dispersion and embedding of antimicrobial agents within polymer matrices, enabling diffusion-driven release profiles suited for sustained antimicrobial action [204].

Laser-based techniques, particularly PLD and MAPLE, can generate controlled nanostructures on thin films, which influence both microbial adhesion and the release dynamics of antimicrobial agents. Nanostructured surfaces can increase the surface area available for interaction with microbial cells, potentially enhancing the contact killing efficiency and the release of antimicrobial agents. Laser ablation itself can be used to create controlled surface patterns and nanostructures on the deposited films [148].

Laser-induced periodic surface structures and nano-patterns can mechanically disrupt bacterial membranes or reduce surface energy to inhibit biofilm formation.

Nanosecond-pulsed laser irradiation generated submicron LIPSS (~0.7 λ) on titanium thin films. The structures featured modulation depths of 70–110 nm, which are within the scale relevant for antimicrobial surface disruption. The authors highlighted how these periodic nano-features can be precisely oriented, offering potential for functional antibacterial coatings [205]. Femtosecond laser pulses induced LIPSS on Al/Ti multilayer thin films. These periodic nano-grooves (320–380 nm) changed the surface chemistry and texture, making the films more resistant to microbial adhesion. Femtosecond laser pulses created low-spatial-frequency LIPSS on Bi thin films, with regular orientation perpendicular to polarization. These structured surfaces exhibited uniformity and repeatability—important for anti-adhesive antimicrobial coatings [206].

Surface nanostructuring also supports osteointegration when combined with bioactive agents for implant coatings. Surface nanostructuring enhances osteointegration by mimicking the scale of extracellular matrix components, thereby promoting cell adhesion, differentiation, and bone matrix formation. When combined with bioactive agents like hydroxyapatite, collagen, or bone morphogenetic proteins, these coatings accelerate tissue integration and implant stabilization [207,208,209].

Covalently immobilized type I collagen on a nanoporous titanium network promoted M2 macrophage polarization, angiogenesis, and osteoblast differentiation, resulting in strong in vivo osseointegration [210]. Hydroxyapatite nanorod surfaces significantly enhanced osseointegration in diabetic rabbit models by improving osteogenesis and angiogenesis [211]. Calcium-rich nanostructured titanium surfaces accelerated bone formation and cell viability compared to conventional HA coatings in vivo [212]. Titanium implants coated with nanoporous silica and bioactive glass particles showed early bone formation and tight bone–implant contact in vivo, enhancing dental implant success [213]. Coatings on titanium using pulsed laser deposition exhibited improved cytocompatibility, hemocompatibility, and long-term bioactivity—ideal for orthopedic applications [214]. Titanium implants coated with protamine/alginate/BMP2 promoted MC3T3-E1 cell differentiation and osseointegration through BMP/Smad pathway activation [215]. In rabbit models, implants with a bioactive glass–nanohydroxyapatite gradient layer showed superior bone-to-implant contact and bone volume compared to HA-only coatings [216]. In a sheep model, this nanostructured titanium surface achieved significantly stronger interfacial shear strength and cortical bone integration than hydroxyapatite-coated controls [217]. CaP nanorod coatings promoted osteoblast differentiation and suppressed inflammation, resulting in stronger bone–implant interfaces in vivo [218].

PLA-HAp-MP4 composites fabricated by laser processing enhanced osteoprogenitor adhesion and differentiation, highlighting the role of protein–nanotopography synergy [219].

Various nanostructuring approaches have been explored to enhance the antimicrobial activity of polymeric composite thin films. For instance, the incorporation of NPs within the polymer matrix can inherently create a degree of surface roughness at the nanoscale. The size, shape, and distribution of these NPs can influence the adhesion of microorganisms to the surface and their susceptibility to the antimicrobial agents. Studies have shown that surfaces with nanoscale features, such as nanopillars or nanodots, can disrupt bacterial cell membranes upon contact, leading to cell death [220].

Sinha et al. observed that laser-deposited SnO_2_ films underwent morphological transitions (from amorphous to crystalline) depending on deposition temperature, affecting both roughness and functional properties like photoluminescence—an indicator of surface reactivity relevant to antimicrobial and sensor applications [221]. Laser-induced surface modification of the deposited films can be employed to create such topographies with high precision. TiO_2_ and ZnO films prepared by PLD showed grain-size-dependent roughness; nanoscale morphology impacted antimicrobial potential and photocatalytic properties [222]. PLD-deposited hydroxyapatite thin films with rough micro/nanotextured surfaces inhibited *E. coli* and *C. albicans* adhesion via contact-based action [223]. Ti and Cu NP-embedded polystyrene films created by cluster beam deposition demonstrated membrane-piercing mechanisms where metal nanoparticles on the surface interact directly with bacterial walls, offering reusability and surface-specific killing for applications such as food packaging or catheter coatings [224].

PLD-created Ti-V-O coatings on titanium surfaces showed improved superhydrophilicity and microstructure, resulting in mechanical inhibition of *S. aureus* colonization by preventing stable cell adhesion. The laser texturing significantly contributed to this bactericidal effect [199].

Magnetron co-sputtered TiO_2_/SiO_2_/Ag nanocomposite coatings on polymeric substrates demonstrated a strong inhibition of bacterial growth via direct surface contact and membrane interaction, as confirmed by significant reductions in *E. coli* viability within the first hour of exposure [225]. The PVP/PVA graphene-doped polymer thin films, though designed for optoelectronics, have surface features and functional groups (e.g., hydroxyls) that can interact with bacterial membranes, suggesting potential for contact-based inactivation mechanisms when adapted to biomedical uses [226].

MAPLE-deposited thin films containing flavonoids and Ag NPs displayed enhanced surface roughness and broad-spectrum antibacterial activity due to the high surface area promoting both contact killing and agent diffusion [153].

Fe_3_O_4_ functionalized with ceftriaxone/cefepime formed nanostructured surfaces that inhibited microbial viability and adhesion, with microscopy images confirming large nanoscale surface area [227].

The high specific surface area of PLGA spheres embedded with Fe_3_O_4_@Gentamicin allowed efficient release of antibiotics and biofilm resistance on silicone and glass surfaces [159].

Furthermore, the nanostructuring of the polymer matrix itself can play a role in antimicrobial activity. For example, electrospinning followed by laser sintering can create porous nanofibrous scaffolds with embedded antimicrobial NPs [228]. The high surface area of these nanofibrous structures can enhance both the interaction with microbial cells and the release of antimicrobial agents. Laser interference lithography is another technique that can be used to create periodic nanopatterns on the surface of the deposited films, which can influence bacterial adhesion and biofilm formation [229].

The advantages of nanostructuring for antimicrobial applications are multifaceted. Increased surface area can lead to a higher density of antimicrobial agents exposed to the microbial environment, enhancing their efficacy. Nanoscale features can also directly interact with microbial cells, disrupting their structure and function. Moreover, controlled surface roughness can influence the wettability of the film, which in turn can affect bacterial adhesion and biofilm formation. PLD coatings with dopants demonstrated how roughness affects bioactivity, bacterial resistance, and bone cell compatibility [230].

Hydrophobic surfaces, for example, have been shown to reduce bacterial adhesion in some cases. Heitz et al. reported that nanostructured surfaces inspired by spider silk showed reduced adhesion to biological materials. While chemical composition also played a role, the nano-rippled surface helped resist unwanted bioadhesion [231]. MAPLE-deposited TiO_2_ nanorods demonstrated smooth morphology with tunable wettability and no degrading surface roughness, making them suitable for photocatalytic and antibacterial applications [232]. Other works have focused on hybrid organic-inorganic systems, such as stearic acid-LDH films deposited via a dual PLD-MAPLE setup, which maintained hydrophobic and bioactive surface characteristics over extended periods [233]. Surface topography and micro/nanoscale structuring of kaolinite films were tuned for hydrophobic or superhydrophilic behavior—key to microbial adhesion resistance in kaolinite [234].

Films incorporating isoflavonoids and antibiotics using MAPLE showed high nanoscale uniformity. The increased surface area minimized microbial adhesion and colonization while maintaining biocompatibility [156].

Nanostructured coatings using magnetite with functionalized surfaces prevented microbial colonization due to increased nanoscale interaction and agent loading capacity. These nanostructured coatings inhibited microbial colonization due to the enhanced contact area between the surface and bacteria while also promoting human cell growth [235].

Voriconazole- and resveratrol-loaded MAPLE films demonstrated active compound release and nanoscale texturing favorable to antimicrobial action [74].

e. Combined influence of environmental factors

One prominent example is the use of MAPLE-fabricated ciprofloxacin–BG–polymer composite coatings, which demonstrated antimicrobial activity against *S. aureus*, *E. coli*, and *P. aeruginosa*. These films inhibited bacterial adhesion due to their nanostructured and hydrophilic surfaces, confirming a surface-mediated contact-killing mechanism in addition to drug release [100].

**Figure 10:**
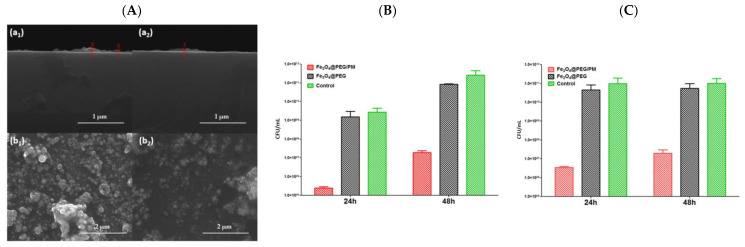
(**A**) Scanning electron microscopy images of Fe_3_O_4_@PEG (**1**) and Fe_3_O_4_@PEG/PM (**2**): (**a**) cross-section; (**b**) coating surface; (**B**) evaluation of biofilm development after 24 and 48 h of incubation in the presence and absence of PEG-based thin film for the *S. aureus* strain; and (**C**) evaluation of biofilm development after 24 and 48 h of incubation in the presence and absence of PEG-based thin film for the *P. aeruginosa* strain [236].

Caciandone et al. developed PEG-functionalized Fe_3_O_4_ (Fe_3_O_4_@PEG) NPs co-loaded with polymyxin B (Fe_3_O_4_@PEG/PM) and deposited via MAPLE onto voice prosthesis surfaces, resulting in thermally stable, nanostructured coatings that dramatically reduced *S. aureus* and *P. aeruginosa* biofilm, highlighting their potential for antibiofilm surface protection (Figure 10). The authors demonstrated that Fe_3_O_4_@PEG NPs co-loaded with polymyxin B inhibit biofilm formation through membrane disruption by polymyxin B, enhanced NP diffusion into the biofilm matrix via PEG functionalization, sustained antimicrobial release from MAPLE-deposited coatings, and reduced microbial adhesion due to anti-fouling PEG surface properties [236].

PLD-synthesized hydroxyapatite coatings from marine origins displayed antimicrobial activity attributed not only to their chemical composition but also to their rough and hydrophilic surfaces, which disrupted microbial adhesion. These films were effective against *E. coli*, *E. faecalis*, and *C. albicans* while supporting mammalian cell growth [223].

Silver oxide thin films, though not polymeric, were shown to inactivate bacteria rapidly via contact-killing supported by visible-light activation and small grain structures—a principle that can be integrated into polymer matrices for combined action [237].

Florea et al. demonstrate that carefully controlled environmental pH and temperature are compatible with silver activity in MAPLE films. The coatings maintained antibacterial activity at physiological pH and temperature. Ag release was balanced for *S. aureus* and *P. aeruginosa*, and the biocompatibility supported osteoblast proliferation [238]. Puiu et al. highlighted how drug or biomolecule interactions affect AgNP antimicrobial performance in polymeric films at biological temperatures. Temperature and biomolecular loading influenced Ag ion bioavailability [239]. EO-loaded nanocoatings showed better selectivity and reduced cytotoxicity under physiological conditions. These laser-deposited coatings exhibited improved hydrophilicity and nanostructure-enhanced release of gentamicin, effectively killing *E. coli*, *S. aureus*, and *C. albicans* [121].

f. Biocompatibility of composite polymeric coatings as an important factor

Beyond antimicrobial efficacy, the biocompatibility of composite polymeric coatings is a notable factor, especially for applications involving direct contact with biological systems, such as medical implants, wound dressings, or food packaging. Biocompatibility refers to the ability of a material to perform with an appropriate host response in a specific application. For antimicrobial coatings, this means that the film must effectively kill or inhibit microbes without causing adverse reactions in human cells, tissues, or the whole environment of the human body. Factors influencing biocompatibility include the release of toxic components from the film, inflammatory responses, and the potential for long-term degradation products. The ideal biocompatible antimicrobial coating should exhibit minimal cytotoxicity, genotoxicity, and immunogenicity, ensuring that it does not provoke an undesirable response from the host’s biological systems [240].

Laser deposition techniques, particularly those using gentler ablation methods like MAPLE, are advantageous in this regard because they can help preserve the integrity of sensitive polymeric and biological components, minimizing the formation of harmful byproducts or alterations to the material’s native structure. The ability to deposit materials at relatively low temperatures and with precise control over film composition and morphology further contributes to achieving desired biocompatible properties. For instance, the controlled incorporation of natural antimicrobial agents or the creation of specific surface topographies that physically deter bacterial growth without relying on leachable toxic compounds can enhance both antimicrobial performance and biocompatibility.

**Figure 11:**
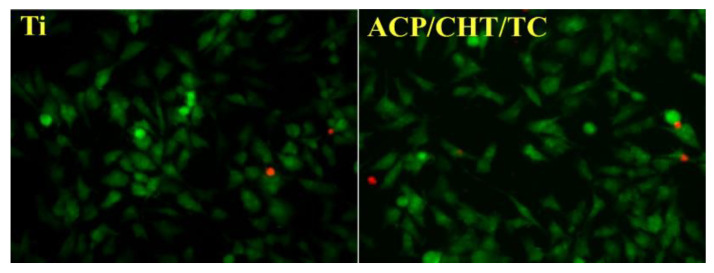
Fluorescence microscopy images of MG63 cells. These images compare cells grown for 24 h on as-deposited composite films (**right**) with those on Ti control disks (**left**). The cells were stained with PI and FDA, and the images were captured at 200× magnification [241].

Achieving a balance between potent antimicrobial action and excellent biocompatibility is a critical design challenge. This requires careful selection of polymers that are inherently non-toxic and biodegradable (if appropriate for the application), as well as antimicrobial agents that are effective at low concentrations and have well-established safety profiles. Furthermore, precise control over the deposition process is essential to ensure the long-term safety and functionality of the composite coating in its intended biological environment. This includes optimizing parameters to prevent the release of unbound or excessively concentrated antimicrobial agents, controlling surface roughness to minimize protein fouling and cell adhesion, and ensuring the stability of the coating over its intended lifespan within the biological system. For example, laser-deposited films (e.g., MAPLE composite films based on amorphous calcium phosphate–chitosan–tetracycline) exhibit excellent cytocompatibility, as demonstrated by in vitro studies (Figure 11) [241].

**Figure 12:**
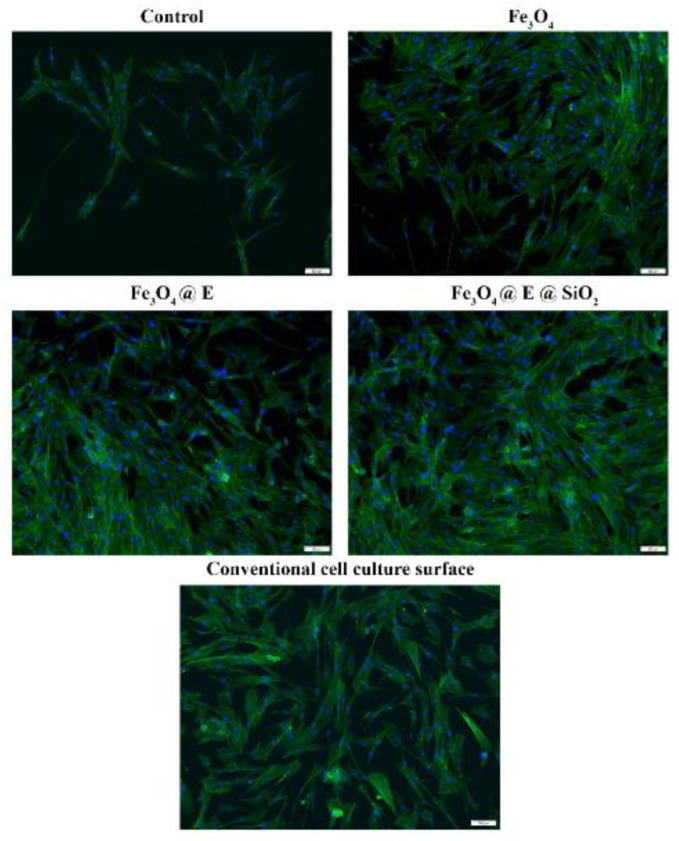
Cytoskeletal organization of human dermal fibroblasts after 48 h on uncoated magnetite and magnetite-, eugenol (E)-, and silica (SiO_2_)-coated magnetite (Fe_3_O_4_)-based nanosystems containing the natural antimicrobial agent coated surfaces with plastic as a control. Actin filaments are shown in green (phalloidin-FITC) and cell nuclei in blue (DAPI). Scale bar: 100 µm [242].

**Figure 13:**
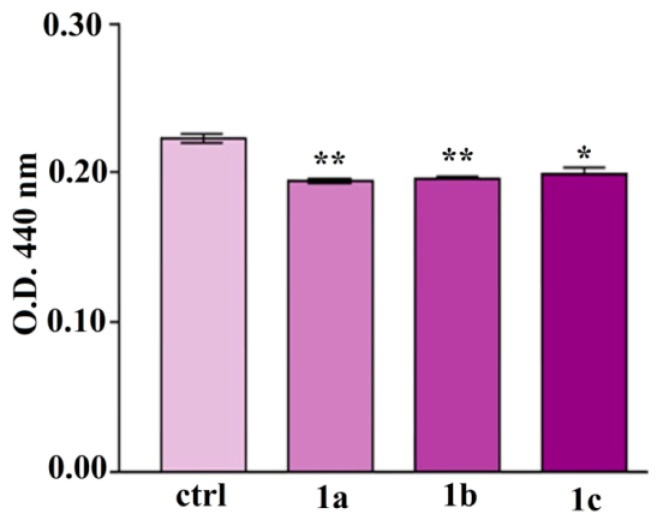
MTT assay quantifying the viability of 3T3-E1 preosteoblasts after 48 h of exposure to bioconstructs 1a, 1b, and 1c, relative to a control. Significant differences from the control are indicated: ** *p* < 0.001 (for 1a and 1b), * *p* < 0.01 (for 1c) [242].

In another study, prepared chitosan-based films have demonstrated excellent biocompatibility with human cells (Figure 12) [242], while PCL/chitosan composite films showed good cytocompatibility with fibroblasts (Figure 13) [243].

Similarly, Ag NPs-decorated poly(l-lactide) films have maintained good cell viability for mammalian cells [244].

Rigorous in vitro and in vivo testing is indispensable to validate the biocompatibility of these advanced polymeric composite films for specific biomedical and food-related applications.

To conclude, the antimicrobial efficacy of laser-deposited polymeric composite thin films is profoundly influenced by key environmental factors, notably surface wettability (hydrophilicity/hydrophobicity) [245], pH [100], temperature [246], and crucial surface nanostructures [247]. Laser deposition techniques offer precise control over these parameters, allowing for tailored film properties that enhance antimicrobial action [80] through mechanisms like direct contact killing [224], optimized agent release [248], and reduced microbial adhesion [199]. Critically, achieving a balance between potent antimicrobial activity and excellent biocompatibility is foremost [249], requiring careful material selection and precise process control to ensure safe and effective performance in diverse biomedical and environmental applications.

### 4.4. Polymeric Antimicrobial Coatings for Combating MDR Pathogens and AMR

Antimicrobial resistance (AMR) is a mounting global crisis, with multidrug-resistant (MDR) bacteria such as methicillin-resistant *S. aureus* (MRSA), vancomycin-resistant *Enterococcus faecalis*, and drug-resistant fungi like *Candida auris* posing significant threats to public health. The World Health Organization has identified these organisms as urgent priorities for novel antimicrobial strategies. Among emerging approaches, polymer-based antimicrobial coatings offer a promising avenue for mitigating pathogen colonization on medical surfaces while minimizing resistance development.

Laser-processed antimicrobial coatings allow precise integration of bioactive compounds, metal nanoparticles (e.g., Ag, Cu, or Se), or functional polymers onto medical surfaces. These coatings exhibit strong antibiofilm and bactericidal effects even against MDR pathogens. A comprehensive work by Popescu et al. highlights the effectiveness of laser-assisted thin coatings on medical devices, particularly noting their application in combating biofilm-associated infections caused by resistant bacteria through surface patterning and nanoparticle integration [14].

Polymeric coatings are highly tunable platforms that can incorporate antimicrobial agents or function inherently as bactericidal surfaces. Several classes of synthetic or natural-origin polymers have demonstrated intrinsic or drug-loaded antimicrobial activity. For instance, ricinoleic acid-based polymers derived from castor oil exhibited broad-spectrum activity against *E. coli*, *P. aeruginosa*, *C. albicans*, *S. aureus*, and notably, MRSA, with excellent biocompatibility and membrane-disruptive effects [250].

Polycarbonates represent another class of biodegradable polymers with exceptional bactericidal performance. Cheng et al. demonstrated that these polymers were more effective than vancomycin in eradicating systemic MRSA infections in a murine model while maintaining low toxicity to human cells [251].

To address biofilm-associated resistance, polymeric coatings incorporating synergistic phytochemicals such as citral and thymol have been successfully deposited on titanium implants. These coatings inhibited MRSA biofilm formation over 60 days and remained non-toxic to peripheral blood mononuclear cells (PBMCs), highlighting their translational potential in orthopedics [252].

Furthermore, halogenated catechol-functionalized polymers have shown >99% killing efficiency against MRSA and MDR *P. aeruginosa*, *A. baumannii*, and *K. pneumoniae* through a membrane-disruptive mechanism. These polymers can be embedded into hydrogels or coatings without losing antimicrobial efficacy and without inducing cytotoxicity [253].

Importantly, novel scaffolds such as 3-((4-hydroxyphenyl)amino)propanoic acid derivatives have exhibited MICs as low as 1–8 µg/mL against MRSA and *Candida auris*, suggesting dual bacterial–fungal activity. These polymer-compatible molecules are promising for broad-spectrum surface applications [254].

Polymeric antimicrobial coatings represent a versatile and potent strategy to address AMR and MDR pathogens, including high-priority species like MRSA and *C. auris*. Their capacity for sustained action, biocompatibility, and resistance avoidance make them ideal candidates for next-generation medical devices and high-touch surfaces in clinical environments.

## 5. Biodegradability and Long-Term Stability of Antimicrobial Polymeric Coatings

For polymeric antimicrobial coatings to be viable in biomedical applications, especially for implants and wound dressings, they must exhibit not only potent antimicrobial activity but also controlled biodegradability and long-term structural stability. These parameters determine the functional lifespan of the coating and its compatibility with biological tissues.

### 5.1. Biodegradability

MAPLE has proven highly effective for the deposition of biodegradable polymers, maintaining both the molecular integrity and functional properties necessary for biomedical applications. This technique is particularly suitable for thermally sensitive polymers such as PLGA, PCL, and PEG, which are frequently used in drug delivery systems, implants, and tissue scaffolds.

#### 5.1.1. Structural Integrity and Functional Preservation

MAPLE allows for the deposition of PEG films that retain their native chemical structure, unlike conventional PLD, which can degrade polymer chains. For instance, PEG films deposited by MAPLE showed nearly identical chemical signatures to their bulk counterparts, as confirmed by FTIR and mass spectrometry, while PLD films suffered from significant molecular breakdown [115].

In a key study, Păun et al. demonstrated that PLGA/PEG coatings deposited by MAPLE exhibited high structural integrity and functional stability across various PEG molecular weights. Low laser fluences preserved chemical bonds, as confirmed by FTIR, and enabled sustained drug release over three weeks in simulated body conditions [255]. Another investigation by Păun and colleagues showed that film integrity declines only above 1 J/cm^2^ fluence. At moderate settings, MAPLE preserved chemical structure and wettability. Excessive fluence increased surface roughness and degraded optical and chemical properties [256]. Further research found that high molecular weight PEG films (PEG 10,000) showed superior hydrophilicity, swelling, and blood compatibility. Despite immersion in PBS, these MAPLE-deposited films retained consistent mass and functional morphology, making them suitable for biomedical use [257].

Cristescu et al. succeeded in depositing metronidazole-linked PEG films via MAPLE without compromising molecular integrity, demonstrated by preserved FTIR spectra and sustained antimicrobial activity. These findings confirm that even chemically modified PEG variants maintain biofunctionality when MAPLE is used [258].

Coatings made of PLCL–PEG–PLCL triblock copolymers were deposited by MAPLE with tunable morphology and roughness. Despite variations in fluence, the main functional groups remained unchanged. These coatings promoted favorable responses from osteoblasts, supporting their utility in bone-contact applications [259].

#### 5.1.2. Tunability of Degradation Rates

Miroiu et al. developed MAPLE-deposited composite coatings of silk fibroin and poly(3-hydroxybutyric-acid-co-3-hydroxyvaleric-acid) (PHBV), which allowed control over degradation rates in simulated body fluid (SBF) at 37 °C. Increasing the PHBV content reduced degradation, enabling customization of drug-release profiles and supporting tissue regeneration applications [260].

Wang et al. showed that varying the substrate temperature during MAPLE deposition of semicrystalline poly(ethylene oxide) films allowed tuning of lamellar thickness and crystal orientation. These structural changes significantly impacted the glass transition temperature and morphological stability, providing a physical mechanism for degradation control [68].

Marturano et al. applied MAPLE to deposit photo-responsive nanocapsule coatings containing thyme oil and coumarin 6. These systems showed light-triggered release and stable adhesion across multiple substrate types, with degradation and release kinetics governed by capsule composition and deposition parameters [52].

Triblock copolymers such as PLCL–PEG–PLCL, deposited by MAPLE, offer control over surface morphology and degradation. These films allow surface tailoring via changes in laser fluence without compromising chemical fidelity, offering customizable degradation profiles essential for osteoblast response and tissue integration [259].

Additionally, MAPLE-deposited PEG/PLGA thin films exhibited degradation behavior dependent on deposition fluence. At higher fluences (>1 J/cm^2^), roughness and hydrophobicity increased, compromising film uniformity and chemistry. Lower fluence and longer deposition times preserved structure, enabling tunable film thickness and sustained degradation [256].

A parametric study involving DNA-CTMA films highlighted that MAPLE enables room temperature deposition without altering conductivity or mobility, crucial for applications requiring delicate polymers and predictable degradation profiles [261].

Similarly, polymer-NPs hybrid coatings using polydopamine and ultra-high molecular weight hydrophilic polymers have shown prolonged antibiofilm activity (over 4 weeks) and mechanical stability through supramolecular interactions, even under dynamic flow conditions [262].

These examples collectively demonstrate the capability of laser deposition to finely modulate degradation rates and surface characteristics through laser fluence, polymer selection, and substrate control, supporting a broad spectrum of biomedical applications, from controlled drug delivery to bioresorbable implants.

#### 5.1.3. Nanocomposite Enhancements

Biodegradability and biofunctionality are further improved by embedding active agents or nanoparticles into the polymer matrix. For example, hydroxyapatite and lactoferrin, when integrated into PEG–PCL matrices, promoted osteoblast adhesion and mineralization while preserving the functional groups post-deposition. This demonstrates MAPLE’s capability for hybrid biodegradable coatings suitable for bone tissue engineering [263].

Similarly, zirconia substrates coated with PEG–PCL by MAPLE, embedded with cyprinol (a fish bile steroid), demonstrated enhanced osseointegration and antibacterial potential for hearing implants, confirming MAPLE’s versatility across hard and soft tissue interfaces [264].

MAPLE was used to deposit polyaniline-lignin coatings embedded with gentamicin-functionalized magnetite (Fe_3_O_4_@GS) for titanium surfaces. These nanocomposites demonstrated strong antimicrobial activity against *E. coli*, *S. aureus*, and *C. albicans*, along with corrosion resistance and improved bone cell compatibility, showing the potential for controlled drug release via magnetic or electric stimuli [121].

Hybrid coatings consisting of PLGA, PEDOT/PSS, cypress oil, and Fe_3_O_4_ NPs were fabricated using MAPLE. These coatings are not only hydrophilic and biocompatible but also antimicrobial and potentially useful in hyperthermia-based anticancer therapies. FTIR confirmed intact transfer of organic components, and the coatings supported osteoblast-like MG-63 cell growth MAPLE deposition of hybrid [151].

Ag NPs-embedded nanofiber composites created for biomedical use show strong antimicrobial properties and wound-healing potential. These composites also offer electroconductivity and can be tuned for smart, responsive behavior in stretchable electronics and sensors [265].

Natural biopolymer nanocapsules based on xyloglucan were successfully loaded with magnetic iron oxide and sulfated quercetin, allowing for targeted drug release while preserving the activity of the molecules. These structures showed superparamagnetic behavior and high colloidal stability, critical for targeted therapy delivery [266].

A doxorubicin-loaded nanocomposite of hydroxyapatite and PLGA demonstrated effective cytotoxicity against osteosarcoma cells. The polymer improved interfacial strength and allowed controlled drug delivery, crucial for cancer therapy targeting bone tissue [267].

### 5.2. Long-Term Structural and Functional Stability

Ensuring long-term integrity of coatings is crucial under physiological, mechanical, and chemical stress. Nanocomposite MAPLE films containing graphene oxide demonstrated significant enhancement in corrosion resistance and oxidative durability when applied to titanium-based implants [268].

Similarly, polydopamine-based coatings combined with ultra-high molecular weight hydrophilic polymers displayed prolonged antibiofilm activity (beyond 4 weeks) due to strong supramolecular interactions, supporting both structural retention and mechanical resilience in dynamic environments [262]. A notable example involves coatings made of PLGA integrated with magnetite nanoparticles and essential oils, which demonstrated both antimicrobial efficiency and gradual biodegradation in physiological conditions [151].

Resonant infrared MAPLE (RIR-MAPLE) also enables thin-film fabrication with minimized photochemical degradation, making it ideal for depositing polymer-nanoparticle blends for long-term use. Coatings can include antimicrobial additives like PEDOT/PSS or essential oils, further enhancing both durability and functional longevity [269].

Additionally, polymer blends such as polyethylene glycol (PEG) and polycaprolactone (PCL) with embedded lysozyme have been used to create MAPLE-deposited coatings. These exhibited sustained antibacterial performance while supporting osteoblast function, indicating a favorable balance between biodegradability and cell compatibility [270].

In one study, coatings developed with graphene oxide embedded in a MAPLE matrix significantly enhanced the corrosion resistance of Ti-based alloys over extended periods in simulated body fluids [268]. This result suggests that appropriate nanocomposite formulations can mitigate degradation without compromising structural integrity.

Similarly, pulsed laser-deposited composite films containing fluorapatite and alumina have shown strong metallurgical bonding and enhanced bioactivity in simulated body fluids, suggesting a promising route to stable, biointegrative coatings [271].

Notably, fluoride-modified hydroxyapatite coatings created via pulsed laser deposition on zirconia implants have significantly improved osseointegration and maintained mechanical integrity in vivo, reinforcing their potential for long-term use [272].

Finally, biodegradable polymer brush coatings like polyphosphoesters are gaining attention for their anti-biofouling and hydrolytic degradation properties [273]. These brushes maintain protein and bacterial resistance while degrading in aqueous environments, offering an excellent strategy for temporary coatings in implants or catheters.

## 6. Challenges and Future Perspectives Regarding Scalability and Industrial Translation of Laser-Based Deposition Techniques

MAPLE has emerged as a valuable method for depositing delicate polymeric and hybrid coatings in biomedical applications, particularly due to its ability to preserve chemical integrity, retain bioactivity, and provide compositional flexibility. Despite these advantages, the scalability and industrial adoption of MAPLE remain constrained by several technical and economic challenges.

One of the primary limitations is the inherently low throughput and deposition rate associated with the MAPLE process. The sublimation of the solvent matrix, which drives material transfer during deposition, is relatively slow and restricts film growth, particularly when thicker coatings are required. Studies have shown that the time-consuming nature of solvent evaporation significantly limits the practicality of MAPLE in high-volume manufacturing environments [274].

Additionally, high capital and operational costs continue to be a significant barrier. The MAPLE technique requires sophisticated laser systems, typically excimer (KrF) or Nd:YAG lasers, along with high-vacuum chambers to ensure film purity and uniformity. While these components offer precise control over film characteristics, they substantially increase the cost per unit area of coated material, making MAPLE less economically competitive than conventional methods like spin coating or spray [275].

Another challenge lies in the complexity of target preparation. The need to create homogeneous frozen suspensions of polymers and bioactive compounds imposes significant labor and technical demands, especially when scaling up the process. The preparation and handling of these cryogenic targets are incompatible with continuous, roll-to-roll production workflows commonly used in industrial environments [274].

Despite these challenges, recent advances suggest promising directions for industrial translation. Hybrid coating strategies that combine MAPLE with more scalable techniques, such as dip coating or spray coating, have been explored. In these hybrid systems, MAPLE is employed to deposit functionally critical bioactive top layers, while bulk film deposition is performed using faster and less costly methods [276].

Furthermore, process optimization through factorial design experiments has demonstrated that tuning deposition parameters such as laser fluence and pulse repetition can enhance both film uniformity and functional performance. Studies involving MAPLE-deposited hybrid oxide coatings have shown improved corrosion resistance and adhesion, indicating a pathway toward scalable and high-performance material systems [33].

The development of RIR-MAPLE adds further potential for scalability by enabling tunable film morphology and reducing photochemical degradation. This technique is especially suited for the fabrication of complex multilayer systems required in advanced biomedical and photonic applications [269].

Moreover, coatings created via MAPLE have demonstrated functional stability after deposition. In particular, long-term antimicrobial activity and mechanical integrity have been preserved even after storage and exposure to simulated physiological conditions. This suggests that, despite current limitations in scale, MAPLE-deposited coatings possess the robustness required for real-world biomedical applications [100].

To address existing bottlenecks and move toward scalable manufacturing, several strategies have been proposed. The design of modular MAPLE systems with multiple nozzles or deposition heads could improve throughput. Efforts to enhance precursor economy, including target recycling and solvent reuse, may reduce operational costs. Most importantly, adapting MAPLE systems and workflows to comply with Good Manufacturing Practice standards will be essential for their integration into clinical and industrial production pipelines [52].

Also, while MAPLE is currently limited in scalability, recent innovations in hybrid deposition, system optimization, and functional validation indicate its strong potential for industrial translation in specialized, high-value biomedical applications [277].

Within these high-value applications, recent trends in PLD/MAPLE-deposited antimicrobial coatings are heavily focused on combating implant-associated infections and antibiotic resistance [276,278]. This includes the deposition of metal NPs, particularly Ag [279] often in conjunction with biocompatible ceramics like magnesium phosphate [280], to achieve both antimicrobial activity and favorable biological responses such as osteoblast proliferation. There’s a growing emphasis on incorporating natural antimicrobial agents and compounds, such as eugenol [281] or isoflavonoids [156], into coatings, often within sophisticated nanosystems or polymeric matrices, aiming for sustained, localized release and reduced development of resistance [282]. Furthermore, advanced coating designs are emerging, including hybrid materials and nanostructured surfaces that not only release antimicrobial agents but also intrinsically prevent microbial adhesion and biofilm formation, offering multifunctional properties for enhanced long-term performance and improved patient outcomes [283].

To address challenges regarding the antimicrobial testing, a set of best practices is proposed:

- Adoption of validated international standards (e.g., ISO 22196, ASTM E2149, ISO 7581) to ensure methodological consistency [284].
- Comprehensive reporting of methodological parameters, including strain identification, CFU/mL inoculum, incubation times, and environmental conditions [285].
- Implementation of multi-method validation (e.g., CFU in conjunction with live/dead staining) to increase confidence in reported outcomes [286].
- Use of dynamic flow systems or ex vivo models that simulate clinically relevant environments, such as saliva or wound exudate [287].
- Inclusion of benchmark controls, such as well-characterized silver-coated or antibiotic-loaded reference materials, to contextualize results [288].

In conclusion, the lack of standardized antimicrobial testing frameworks remains a significant bottleneck in the development and clinical translation of laser-deposited antimicrobial coatings. Harmonization of test protocols and improved transparency in experimental reporting will be critical in advancing this promising field.

## 7. Conclusions

This short review highlights the potential and current advancements in the use of polymeric composite thin films fabricated via laser techniques for antimicrobial applications. The key takeaways from the study are as follows:

- Laser-based deposition methods such as PLD and MAPLE enable precise, clean, and efficient fabrication of antimicrobial coatings with controllable properties. The choice between MAPLE and PLD depends on factors such as the nature of the polymer and antimicrobial agent, desired film properties (thickness, morphology, uniformity), substrate material, and considerations of scalability and cost-effectiveness. PLD offers higher deposition rates but risks thermal damage; MAPLE preserves bioactivity but has lower throughput.
- Polymeric matrices embedded with antimicrobial agents (e.g., metal NPs, antibiotics, natural compounds) enhance surface functionality and broaden the antimicrobial spectrum. Ag NPs provide rapid bactericidal effects but raise cytotoxicity concerns; antibiotics (e.g., ciprofloxacin) offer targeted action but may induce resistance.
- Hybrid systems (e.g., Ag NPs + chitosan) balance efficacy and safety, with MAPLE being optimal for delicate biomolecules.

Future prospects involve optimizing material combinations, enhancing biocompatibility, and scaling up production for commercial and clinical use.

## Figures and Tables

**Table 1:** Advantages and disadvantages of laser techniques for polymeric composite thin films.

Technique	Overview	Advantages	Disadvantages	Reference
MAPLE	Designed for organic materials and polymers. A frozen matrix containing the polymer is irradiated with a laser, ejecting material onto a substrate.	Ideal for delicate polymers and bioactive agents Minimal thermal degradation Preserves chemical integrity and morphology Good for biocompatible and drug-loaded films	Lower deposition rate Often requires solvent removal Uniformity can be substrate-dependent	[32]
PLD	A high-power laser ablates a solid target (can be polymeric or composite) in a vacuum or a gas atmosphere.	High deposition rate Suitable for metal oxides and hybrid systems Stoichiometric material transfer Used with polymers and nanoparticle composites	Risk of thermal damage Particulate emission Non-uniform deposition over large areas	[33]

**Table 2:** Deposition parameters influencing PLD and MAPLE techniques.

Parameter	Effect on Film Properties	Relevant Technique(s)	Reference
substrate temperature	Modulates adhesion, crystallinity, and solvent evaporation; critical for morphology control	PLD	[68]
background gas pressure	Influences plasma plume dynamics and energy transfer; affects film density and composition		[69]
matrix solvent	Should absorb laser well and evaporate cleanly; affects analyte preservation and plume quality	MAPLE	[70]
active substance concentration	Low concentrations reduce aggregation and enhance uniform molecular transfer		[71]
target-substrate distance	Controls kinetic energy and flux; affects uniformity and deposition rate	PLD, MAPLE	[69]
laser wavelength	Affects absorption and ablation efficiency; UV preferred for polymers to minimize damage		[33]
pulse duration and repetition rate	Short pulses reduce thermal damage; repetition rate influences film growth rate and roughness		[72]
laser fluence	Controls amount of material ablated; if too high, may cause damage; if too low, reduces efficiency		[73]

**Table 3:** Summary of variability and associated mechanisms.

Pulse Duration	Primary Mechanism(s)	Thermal Impact	Common Applications	Refs.
Ns	Thermal melting and vaporization; plasma shielding	Significant	PLD, MAPLE, bulk material removal (cutting, welding)	[79,94]
Ps	Hybrid (transition from thermal to non-thermal); reduced electron–phonon coupling time	Minimized	High-precision micromachining, LIPSS (less common than fs)	[79,95]
Fs	Non-thermal (Coulomb explosion, phase explosion, multiphoton ionization)	Negligible	LIPSS, ultra-precision micromachining, transparent material processing, medical applications	[79,95]

**Table 4:** Advantages and disadvantages of polymer matrices and NPs in antimicrobial coatings.

Component	Advantages	Disadvantages	Ref.
Polymer Matrices	-Biocompatible (e.g., PLGA, chitosan)	-Limited thermal stability (degradation in PLD)	[103].
	-Tunable drug release kinetics	-Variable mechanical strength	[104]
	-Flexible substrate adhesion	-Potential cytotoxicity (e.g., PVP at high doses)	[105]
NPs	-High surface-area-to-volume ratio (e.g., Ag NPs)	-Aggregation risks	[106]
	-Broad-spectrum antimicrobial activity	-Potential cytotoxicity (e.g., Cu NPs)	[107]
	-Synergistic effects with polymers	-Complex synthesis and functionalization	[108]

**Table 5:** Commonly used organic antimicrobial agents.

Category	Compound	Application In Thin Films	Reference
Antibiotic	Gentamicin	Incorporated in poly(sebacic anhydride) films via MAPLE; active against *E. coli* and *S. aureus*	[147]
	Kanamycin	Functionalized onto Fe_3_O_4_ NPs; MAPLE-deposited films inhibit microbial adhesion and biofilm formation	[148]
	Ceftriaxone/Cefuroxime	Loaded into HAp/PLGA coatings; prevent *E. coli* adhesion and biofilm development on bone implants	[149]
	Ciprofloxacin	Embedded in bioglass–polymer composite coatings for titanium implants with antibacterial properties	[100]
	Doxycicline	Embedded in bioglass–polymer composite coatings for stainless steel implants with antibacterial properties against *S. aureus* and *E. coli*	[150]
Essential oils	Cypress oil	Combined with Fe_3_O_4_ nanoparticles in PLGA coatings; antimicrobial against *S. aureus*, *E. coli*, and *C. albicans*	[151]
	Tea Tree Oil (Terpinen-4-ol)	Used in plasma-polymerized films; inhibits *P. aeruginosa* biofilm formation	[152]
Natural powders	Propolis	Incorporated into bioactive coatings; shown to enhance antimicrobial performance and healing	[153]
	Neem	Combined with bioglasses for antimicrobial activity against *S. aureus*	[154]
Natural extract	Salicylic acid	Combined with silica/magnetite layers; contributes to antibiofilm properties in laser-deposited coatings	[155]

**Table 6:** Commonly used antimicrobial testing methods and their limitations.

Method	Strengths	Limitations	Reference
Zone of Inhibition (ZOI)	Simple, rapid screening tool	Limited to diffusible agents; not applicable for contact-killing surfaces	[160]
Colony Forming Unit (CFU)	Quantitative measurement of viable bacteria	Time-consuming; may miss VBNC cells; highly variable based on growth phase and incubation conditions	[161]
Live/Dead Staining (SYTO9/PI)	Direct imaging of viable vs. compromised cells	Requires fluorescence microscopy; possible overestimation due to temporary permeability changes	Not standardized
Metabolic Assays (MTT, resazurin)	Rapid, adaptable to high-throughput screening	Indirect measure; affected by material-specific interference	Not always reported
Biofilm Quantification (CV Assay)	Useful for assessing biofilm biomass over time	Cannot distinguish live vs. dead cells; limited sensitivity	[162]

**Table 7:** Mechanistic influence of surface and environmental factors on contact-killing antibacterial activity.

Factor	Mechanism	Influence on Contact Killing	References
Hydrophobicity	Lipid membrane interaction	Moderate hydrophobicity = ↑ membrane rupture	[185]
Hydrophilicity	Reduced adhesion, biofilm prevention	↑ Water-wettability = ↑ contact area	[186]
pH	Surface charge and membrane binding	↑ Protonation at low pH = ↑ cationic interaction	[187]
Temperature	Membrane fluidity and surface energy	↑ Temperature = ↑ membrane fluidity = ↑ vulnerability to rupture	[188]
Nanostructures	Mechanical puncture or trapping	↑ Nanoscale roughness = ↑ disruption efficiency	[189]

## Data Availability

The contributions presented in the study are included in the article; further inquiries can be directed to the corresponding author.
